# The efflux system CdfX exports zinc that cannot be transported by ZntA in *Cupriavidus metallidurans*

**DOI:** 10.1128/jb.00299-24

**Published:** 2024-10-30

**Authors:** Vladislava Schulz, Diana Galea, Grit Schleuder, Philipp Strohmeyer, Cornelia Große, Martin Herzberg, Dietrich H. Nies

**Affiliations:** 1 Martin-Luther-University Halle-Wittenberg, Institute for Biology/Microbiology, Halle (Saale), Germany; 2 Department of Analytical Chemistry, Helmholtz Centre for Environmental Research – UFZ, Leipzig, Germany; NCBI, NLM, National Institutes of Health, Bethesda, Maryland, USA

**Keywords:** *Cupriavidus metallidurans*, zinc, zinc transport, CDF proteins

## Abstract

*Cupriavidus metallidurans* is able to survive exposure to high concentrations of transition metals, but is also able to grow under metal starvation conditions. A prerequisite of cellular zinc homeostasis is a flow equilibrium combining zinc uptake and efflux processes. The mutant strain ∆e4 of the parental plasmid-free strain AE104 with a deletion of all four chromosomally encoded genes of previously known efflux systems ZntA, CadA, DmeF, and FieF was still able to efflux zinc in a pulse-chase experiment, indicating the existence of a fifth efflux system. The gene *cdfX*, encoding a protein of the cation diffusion facilitator (CDF) family, is located in proximity to the *cadA* gene, encoding a P-type ATPase. Deletion of *cdfX* in the ∆e4 mutant resulted in a further decrease in zinc resistance. Pulse-chase experiments with radioactive ^65^Zn(II) and stable-isotope-enriched ^67^Zn(II) provided evidence that CdfX was responsible for the residual zinc efflux activity of the mutant strain ∆e4. Reporter gene fusions with *cdfX-lacZ* indicated that the MerR-type regulator ZntR, the main regulator of *zntA* expression, was responsible for zinc- and cadmium-dependent upregulation of *cdfX* expression, especially in mutant cells lacking one or both of the previously characterized efflux systems, ZntA and CadA. Expression of *zntR* also proved to be controlled by ZntR itself as well as by zinc and cadmium availability. These data indicate that the *cdfX-cadA* region provides *C. metallidurans* with a backup system for the zinc-cadmium-exporting P-type ATPase ZntA, with CdfX exporting zinc and CadA cadmium.

**IMPORTANCE:**

Bacteria have evolved the ability to supply the important trace element zinc to zinc-dependent proteins, despite external zinc concentrations varying over a wide range. Zinc homeostasis can be understood as adaptive layering of homeostatic systems, allowing coverage from extreme starvation to extreme resistance. Central to zinc homeostasis is a flow equilibrium of zinc comprising uptake and efflux reactions, which adjusts the cytoplasmic zinc content. This report describes what happens when an imbalance in zinc and cadmium concentrations impairs the central inner-membrane zinc efflux system for zinc by competitive inhibition for this exporter. The problem is solved by activation of Cd-exporting CadA or Zn-exporting CdfX as additional efflux systems.

## INTRODUCTION

**Fig 1:**
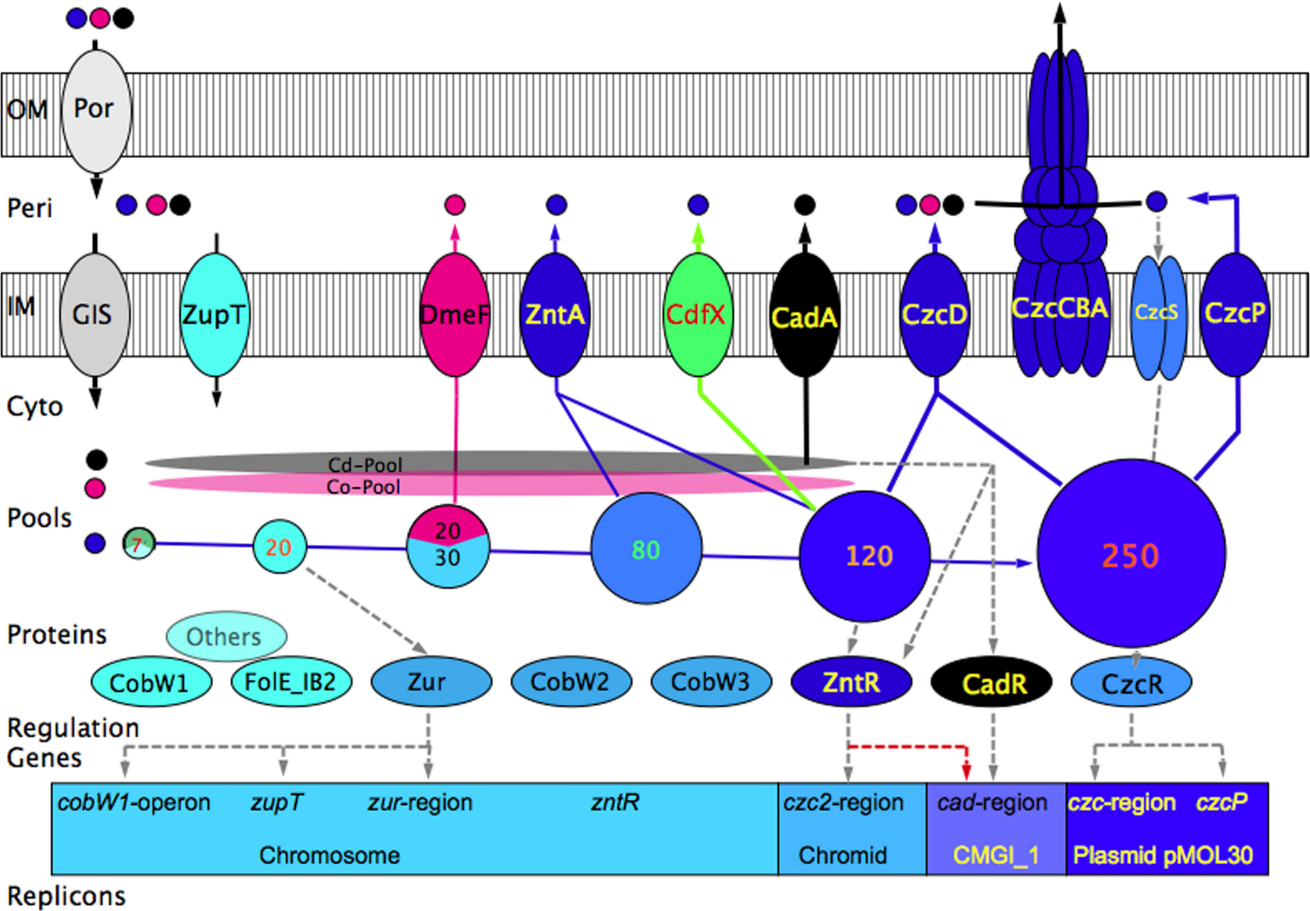
CdfX contributes to zinc homeostasis in *C. metallidurans*. The schematic shows the response of *C. metallidurans* to the continuum of increasing zinc concentrations and highlights the function and regulation of the novel CDF protein, CdfX. The cell is depicted from the outer membrane (OM) via the periplasm, inner membrane (IM), cytoplasm, the metal pools, main proteins involved, regulatory connections (gray dashed lines), and the genes and the replicons they are located on. The membranes contain transport proteins such as the porins and general import systems (GIS). The colors are used to indicate increasing zinc concentrations (from light to dark blue) and the factors acting at the respective concentrations, as well as cobalt (magenta) and cadmium (black). Light green indicates new findings. The circles represent the pool sizes of zinc or zinc-substituting cobalt within the cellular content indicated in thousand atoms per cell. A sector in magenta (third pool from the left) shows cobalt substituting for zinc at low zinc concentrations (9), one in green (first pool to the left) the zinc portion in the important zinc-dependent protein, RpoC (10), the beta-prime subunit of the RNA polymerase, which accounts for about two-thirds of the 7,000 Zn atoms present in strongly zinc-starved cells. Details in the text.

*Cupriavidus metallidurans* CH34 is a metal-resistant beta-proteobacterium that can be found in sites rich in transition metals such as zinc deserts or auriferous soils (1–4). Its genome is composed of a chromosome, a chromid and two plasmids that carry many metal-resistance determinants (4–6), including the cobalt-zinc-cadmium resistance determinant *czc* and that for cobalt and nickel, *cnr* (7). The most important gene products of these determinants are large hetero-multimeric transmembrane protein complexes, such as CzcCBA, which export their substrate cations from the periplasm to the environment outside of the cell (8). Together with periplasmic metal-binding proteins and additional metal efflux pumps of the inner membrane, these transenvelope pumps adjust the periplasmic metal ion concentration and composition so that the subsequent import into the cytoplasm does not lead to toxic concentrations in this cellular compartment (Fig. 1).

The metal ion concentration and composition are determined through uptake into the cytoplasm by at least nine different import systems (Fig. 1, GIS for general import systems) plus ZupT for zinc ions (11–13), together with the activities of specific efflux pumps, such as the P-type ATPases ZntA for zinc, CadA for cadmium, PbrA for lead, CupA for copper, and the cation diffusion facilitators (CDF proteins) DmeF for cobalt and FieF for iron, whose function is removal of surplus metal ions from the cytoplasm to the periplasm (14–22). Together with metal-buffering compounds, for example, glutathione and polyphosphate, in the cytoplasm and directed by two CobW-proteins of the COG0523 family of metal-binding GTPases, this leads to a flow equilibrium of the concentration of the single metal cations such as Zn(II), which adjusts the cytoplasmic metal ion content by this combination of controlled import and export reactions (23). As expected, metal uptake systems, metal-complexing compounds, the CobWs and metal efflux systems are required for the full function of this flow equilibrium (Fig. 1) (23). Surprisingly, the ∆e4 mutant (∆*zntA ∆cadA ∆dmeF ∆fieF*) of the plasmid-free *C. metallidurans* strain AE104 was still able to perform a residual zinc efflux reaction, which indicated the presence of another, so far uncharacterized, efflux system.

The P_IB2_-type ATPases ZntA and CadA transport Zn(II) and Cd(II) *in vitro* with similar kinetic parameters and substitute each other (18, 19). Expression of *zntA* is under control of the MerR-type protein ZntR. This regulator and its binding to the *zntAp* promoter have been characterized *in vitro* (20). ZntR is not different from other MerR-type metal regulators with respect to its function as repressor-activator (24–29).

Expression of the *cadA* gene is under the control of the MerR-type regulator CadR (20). Both genes are adjacent and inversely oriented on the chromosome, located on the CMGI_1 genomic island (30, 31) and transcribed from RpoD-dependent promoters (32) (Fig. S1). The transcriptional start sites of *cadAp* and *cadRp* overlap and are separated by 6 base pairs (bp). Co-transcribed with, and downstream of, *cadA* are *cadC* and the gene locus tag Rmet_2305; downstream of *cadR* are the genes with locus tags Rmet_2300 and Rmet_2299. Transcription continues downstream of Rmet_2299 as antisense-RNA to non-expressed genes on the opposing DNA strand. The *cadA* gene was deleted from a position 10 bp upstream of *cadR* to 11 bp upstream of the *cadA* stop codon so that the RpoD-dependent *cadRp* promoter upstream of *cadR* was also deleted (32). Consequently, Rmet_2299 is only expressed from the promoter directly upstream of its open reading frame (Fig. S1) in all ∆*cadA* strains; however, it is expressed from this promoter and from the *cadRp* promoter in all other strains.

With changing metal concentrations in the environment, the genes in the neighborhood of *cadA* are differentially upregulated (Fig. S1), while the 3-fold upregulation of Rmet_2300 is below the threshold for a response to changing metal availability (33). CadC is a predicted pro-lipoprotein signal peptidase, while Rmet_2300 and Rmet_2305 are small cytoplasmic proteins with unknown functions. Rmet_2299 encodes a predicted membrane protein with six transmembrane alpha-helices (Fig. S1), and is a member of the CDF protein family (15). In comparison to two other CDF proteins in the plasmid-free *C. metallidurans* strain AE104, the cobalt exporter DmeF and the iron exporter FieF, Rmet_2299 was predicted by AlphaFold (34) to have a more compact membrane domain and a much smaller cytoplasmic domain. DmeF and FieF contain a cytoplasmic domain with beta-sheet structure, which is needed for dimerization as shown for the FieF ortholog YiiP (35–38). Both proteins also contain an intrinsically disordered domain with an accumulation of metal-binding amino acid residues, which are probably metal-binding sites. As a consequence, DmeF is a protein with the most metal-binding sites in the proteome of *C. metallidurans* (39, 40). The dimerization and the intrinsically disordered metal-binding sites are absent in the Rmet_2299 protein, but this protein is clearly a member of the CDF protein family. Consequently, it was named “CdfX.” This study shows that CdfX is involved in zinc homeostasis in *C. metallidurans* and is part of the zinc-cadmium regulatory network in this bacterium. This adds a further layer of complexity to zinc homeostasis in *C. metallidurans* between the flow equilibrium at ambient zinc concentrations and zinc resistance mediated by the transenvelope efflux systems.

## RESULTS

### CdfX is involved in zinc homeostasis

**Fig 2:**
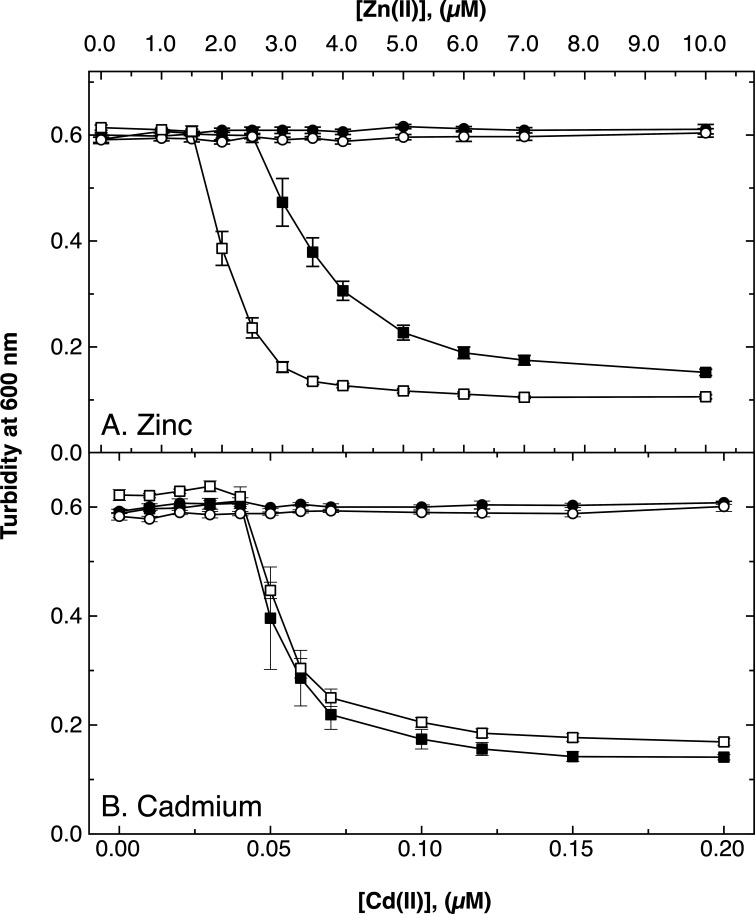
Resistance of a ∆e4 ∆*cdfX* mutant to zinc and cadmium. A dose–response experiment in moderate zinc (mZn) TMM is shown (*n* = 7). Compared are the AE104 parent cells (circles) and its ∆e4 (∆*zntA ∆cadA ∆dmeF ∆fieF*) mutant (squares) without (filled symbols) or with an additional ∆*cdfX* deletion (open symbols). The incubation time of the main culture was 20 h with shaking at 30°C in 96-well plates after a 10-fold dilution of a 24-h preculture in the same growth medium.

The *cdfX* gene was deleted in the plasmid-free *C. metallidurans* strain AE104 and in the isogenic ∆e4 (∆*zntA ∆cadA ∆dmeF ∆fieF*) quadruple mutant of this strain. These strains were cultivated in standard Tris-buffered mineral salts medium (TMM). Deletion of *cdfX* in strain AE104 had no effect on zinc or cadmium resistance up to 10 µM Zn(II) or 200 nM Cd(II), respectively (Fig. 2), but clearly caused a further decrease of zinc resistance in the ∆e4 strain from an IC_50_ of about 4 µM to just 2 µM Zn(II). Significant differences in cadmium resistance between strains ∆e4 and ∆e4 ∆*cdfX* could not be determined (Fig. 2B). The IC_50_ for both strains was around 60 nM Cd(II). Involvement of CdfX in cadmium resistance could not be demonstrated; however, this newly identified CDF protein from *C. metallidurans* appears to be involved in zinc homeostasis when the other zinc efflux systems are absent.

### CdfX affects the flow equilibrium of zinc

The effect of a *cdfX* deletion on zinc homeostasis was also analyzed in a pulse-chase experiment, performed as published (23). The ∆e4 ∆*cdfX* mutant and its ∆e4 parent were cultivated in ambient zinc (aZn) medium adjusted to 200 nM Zn(II). Moreover, the cells were also incubated in low-zinc, low-magnesium medium, which increased zinc uptake sevenfold compared to ambient zinc medium or medium with a lower zinc concentration but 1 mM magnesium chloride (23).

**Fig 3:**
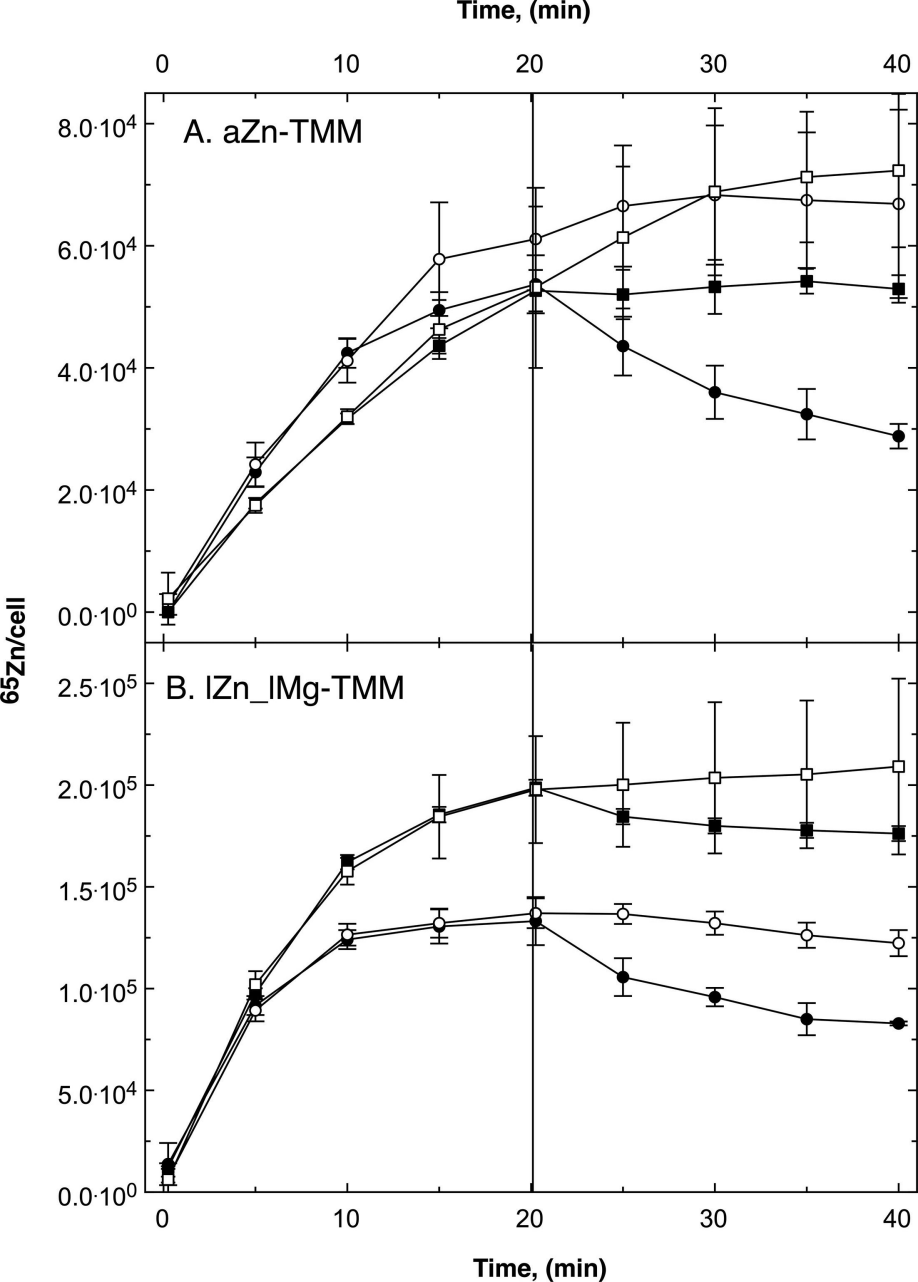
Pulse-chase experiment with ∆e4 and its ∆*cdfX* mutant. The cells were cultivated in TMM containing 200 nM Zn(II) (aZn-TMM, Panel A) or in a low metal medium without added zinc and 100 µM Mg(II) instead of 1 mM (lZn_lMg-TMM, Panel B). A preculture of the same medium was used. At a turbidity of 150 Klett units, the cells were harvested by centrifugation, suspended in an equal volume of 10 mM Tris-HCl (pH 7.0) and stored on ice until needed, but not for longer than a few hours. To 6 mL of this cell suspension, 2 g/L Na gluconate was added before the experiment. The uptake reaction (pulse) was started by addition of 12 µL ^65^Zn (500 µM, 12 µCi, 1 µM final concentration). The cells were incubated with shaking at 30°C. Samples of 500 µL were collected by filtration (0.2 µm pore size), washed two times with 5 mL ice-cold wash solution (50 mM Tris-HCl pH 7.0, 50 mM EDTA) and radioactivity was measured in a scintillation counter. After 20 min (bar), non-radioactive Zn(II) was added (chase) to a final concentration of 100 µM (closed symbols), not (open symbols) and sampling was continued. Strain ∆e4 (circles) and its ∆*cdfX* mutant (squares) were compared. Three biological repeats are shown with deviation bars given.

The cells were incubated with 1 µM of radioactive ^65^Zn to measure zinc uptake and subsequently chased with 100 µM non-radioactive zinc (Fig. 3A). A control experiment was also done with the chase. The initial ^65^Zn uptake velocity *v*_up_, the ^65^Zn-content after 20 min *C*_20_ (determined as the quotient *C*_max_/*C*_20_ of the extrapolated final zinc content after the uptake phase, divided by *C*_20_), the initial net efflux rate *v*_ef_ and the quotient *C*_40_/*C*_20_ after 40 and 20 min were calculated, with the last two values for the chased and the non-chased control also being determined (Table 1). In cells grown under zinc-replete conditions, the initial uptake velocity *v*_up_ between mutant ∆e4 ∆*cdfX* and parent ∆e4 was similar and the cells also reached similar zinc levels within the uptake period (Table 1; Fig. 3A). The *C*_max_/*C*_20_ values of around 3 indicated that both strains had not reached a saturation maximum of zinc during this time. While the parent ∆e4 clearly exported ^65^Zn again when chased, the ^65^Zn content of the ∆e4 ∆*cdfX* mutant remained at the same level. After 40 min, the ∆e4 parent had lost half of its ^65^Zn atoms per cell while the ∆e4 ∆*cdfX* mutant retained its zinc ions. There was no net efflux of radioactive zinc in the ∆e4 ∆*cdfX* mutant compared to the ∆e4 parent but the non-chased ∆e4 ∆*cdfX* control cells continued to import ^65^Zn, while the chased cells did not. This may indicate a residual efflux activity of the ∆e4 ∆*cdfX* cells with a rate similar to that of the import rate for ^65^Zn, which was still present in the uptake buffer despite its dilution with a 100-fold higher concentration of non-radioactive zinc.

**TABLE 1:** Results of the pulse-chase experiments with *C. metallidurans* ∆e4 and ∆e4 ∆*cdfX^a^*

	Uptake			Chase		Pulse continued	
Strain	*v*_up_, (1 /s)	*C*_20_, 1000 Zn/cell	*C*_max_/*C*_20_	*v*_eff_, (1 /s)	*C*_40_/*C*_20_	*v*_eff_, (1 /s)	*C*_40_/*C*_20_
	aZn-TMM						
∆e4	97 ± 0	57 ± 5	2.26	26.9 ± 0.1	50.2 ± 3.5%	−4.1 ± 0.0	116.5 ± 26.9%
∆e4 ∆*cdfX*	66 ± 2	53 ± 0	3.29	−0.9 ± 0.0	100.0 ± 4.3%	−14.3 ± −0.1	136.6 ± 23.7%
	lZn-lMg-TMM						
∆e4	667 ± 61	135 ± 3	1.26	49.1 ± 0.2	61.3 ± 0.7%	14.4 ± 0.0	90.6 ± 4.7%
∆e4 ∆*cdfX*	493 ± 35	198 ± 1	1.60	18.0 ± 0.0	88.9 ± 1.9%	−9.1 ± 0.0	105.5 ± 21.8%

^*a*^ These pulse-chase experiment with ^65^Zn measured (23): (i) the initial zinc uptake velocity *v*_up_(0) at *t* = 0; (ii) the cellular ^65^Zn content *C*_20_ at the end of the uptake period (time point represented in Fig. 3 by the horizontal bar); (iii) the extrapolated maximum zinc content after the uptake period *C*_max_; (iv) the efflux velocity *v*_eff_ at the beginning of the chase at 20 min; and (v) the final zinc content *C*_40_ at the end of the chase period (Fig. 2, *t* = 40 min). To obtain these data, the uptake phase up to 20 min of the pulse-chase experiment was adapted to the equation *C*(*t*) = *C*_max_ · *t*/(*K*_*t*_ + *t*) using the Lineweaver-Burk-like plot 1 /*C*(*t*) = 1/*C*_max_ + *K*_*t*_/*C*_max_ · 1 /*t*. The first deviation by time of the equation *C*(*t*) = *C*_max_ · *t*/(*K*_*t*_ + *t*) was d*C(t*)/d*t* = *C*_max_ · *K*_*t*_/(*K*_*t*_ + *t*)^2^. At *t* = 0, this gave the initial uptake rate *v*_up_(0) = *C*_max_/*K*_*t*_. After the chase after 20 min, the cell-bound zinc content was modeled by the decay function *C*(*t*) = *C*_o_ · e^(−τ . *t*) using the plot ln *C*(*t*) = ln *C*_o_ −τ · *t*. The first deviation by time of the equation *C*(*t*) = *C*_o_· e^(−τ · *t*) was d*C*(*t*)/d*t* = −τ · *C*_o_ · e^(−τ · *t*). And at *t* = 0, this value was the initial net efflux rate *v*_ef_(0) = −τ · *C*_o_. In contrast to the initial uptake rate that was no net rate because the cells did not contain ^65^Zn at *t* = 0, *v*_ef_(0) was a net rate and the result of the real efflux rate after chase minus the rate of ^65^Zn re-import at this time.

In cells cultivated under zinc- and magnesium-starvation conditions (Fig. 3B), the ∆e4 ∆*cdfX* cells and their ∆e4 parent imported ^65^Zn with a similar rate, which was seven times higher than the uptake rate of zinc replete cells, confirming earlier observations (23, 41). Loss of neither CdfX, nor the four other efflux systems, affected this specific difference between zinc-replete and metal-starved cells. Metal-starved ∆e4 ∆*cdfX* cells attained a higher zinc level after 20 min than their parent ∆e4 (Fig. 3; Table 1). The quotient *C*_max_/*C*_20_ indicated that a flow equilibrium had been reached in the ∆e4 strain and nearly the same quotient was also determined for its ∆*cdfX* mutant. In the subsequent chase phase, ∆e4 cells demonstrated efflux. Although the ∆e4 ∆*cdfX* cells also exhibited an initial efflux rate of 1/3 of that of their parent, the data points for the chased cells were within the standard deviation of the control. Thus, clear evidence for or against a residual efflux activity in the ∆e4 ∆*cdfX* strain could not be categorically stated; nonetheless, the findings demonstrated that CdfX is responsible for the net zinc-efflux activity of the ∆e4 strain. CdfX thus represents the fifth zinc efflux system in *C. metallidurans*; however, the existence of a further sixth system cannot be excluded.

To provide experimental evidence for this other zinc efflux system, the pulse-chase experiments with radioactive ^65^Zn were accompanied in parallel with experiments using isotope-enriched stable ^67^Zn for the uptake reaction. This allowed us to differentiate between the two zinc pools, ZP1, which has a natural isotope composition (28) and is present in the cells at the start of the experiment, and using ^67^Zn, pool ZP2, which accumulates during the uptake reaction (23). Although cultivated in the presence of sufficient zinc, the ∆e4 ∆*cdfX* cells contained only 20% of the initial zinc content in ZP1 compared to their ∆e4 parent cells (Table 2). The ∆e4 cells possessed 84,000 Zn ions per cell, which is similar to the level in its parent strain AE104 (10, 13, 23, 42). This means that the four efflux systems ZntA, CadA, DmeF, and FieF did not influence the accumulation of zinc at ambient zinc concentrations but CdfX did. When 1 µM ^67^Zn was added, ∆e4 and ∆e4 ∆*cdfX* both contained about 110,000 Zn per cell, which went into ZP2 without much loss of zinc from the initial zinc pool, ZP1. The ∆e4 ∆*cdfX* cells accumulated a much higher number of ^67^Zn ions during the uptake phase than their parent ∆e4. Both strains lost 2/3 of the zinc in ZP2 during the subsequent chase, which indicated that both were able to exchange ^67^Zn against non-isotope enriched zinc. This means that the loss of CdfX in strain ∆e4 resulted in an upregulation of zinc import.

**TABLE 2:** Summary of the experiments with stable ^67^Zn that accompanied the pulse-chase experiments with radioactive ^65^Zn^*a*^

Strains	Initial 10^3^ Zn			10^3^ Zn after pulse			10^3^ Zn after chase (0.1 mM)		
Medium	ZP1	ZP2; %ZPt	ZP1 + ZP2	ZP1	ZP2; %ZPt	ZP1 + ZP2	ZP1	ZP2; %ZPt	ZP1 + ZP2
Ambient Zn									
∆e4	84.4 ± 3.1	<0	84.2 ± 3.1	78.5 ± 4.8	23.2 ± 0.3; 23%	102 ± 5	502 ± 49	7.2 ± −0.3; 1%	509 ± 49
∆e4 ∆*cdfX*	16.7 ± 9.6	<0	16.6 ± 9.5	17.5 ± 8.4	97.7 ± 26.5; 85%	115 ± 35	800 ± 146	36.8 ± 14.1; 4%	837 ± 160
Low Zn and Mg									
∆e4	7.6 ± 0.8	0.0	7.6 ± 0.8	8.1 ± 0.3	91.6 ± 5.1; 92%	99.7 ± 5.5	767 ± 100	37.1 ± −0.4; 5%	805 ± 99
∆e4 ∆*cdfX*	7.3 ± 1.0	<0	7.3 ± 1.0	8.3 ± 0.8	89.2 ± 22.9; 92%	97.4 ± 23.6	688 ± 241	50.9 ± 17.4; 7%	739 ± 259

^*a*^ The cells of the indicated *C. metallidurans* strain were incubated in Tris-buffered mineral salts medium adjusted to 200 nM Zn(II) (ambient zinc) or the same medium without trace element solution SL6 and 0.1 mM Mg(II) instead of 1 mM Mg(II) (low Zinc and Mg). Zinc coming from SL6 or contaminations was in the natural isotope composition. These cells were incubated with 1 µM enriched stable ^67^Zn for 20 min (pulse) and subsequently chased with 100 µM Zn(II) with the natural isotope composition again. The zinc pools ZP1 and ZP2 were calculated from the ICP-MS measurements and ZPt = ZP1 + ZP2 was determined. The ∆e4 values and been published (23) and were obtained under the same condition as those of the ∆e4 ∆*cdfX* cells.

In metal-starved cells (Table 2), the initial zinc content was similar and very low due to low zinc concentrations in the growth medium. Both strains contained 90,000 Zn ions per cell after the uptake phase and exported ^67^Zn during the subsequent chase period. Both were still able to perform zinc export. This indicated that indeed one more efflux system must exist in the ∆e4 ∆*cdfX* mutant. The data also demonstrated that CdfX had a role in control of the accumulation of zinc at ambient zinc concentrations.

### Expression of the *cdfX* gene is influenced by ZntR

**Fig 4:**
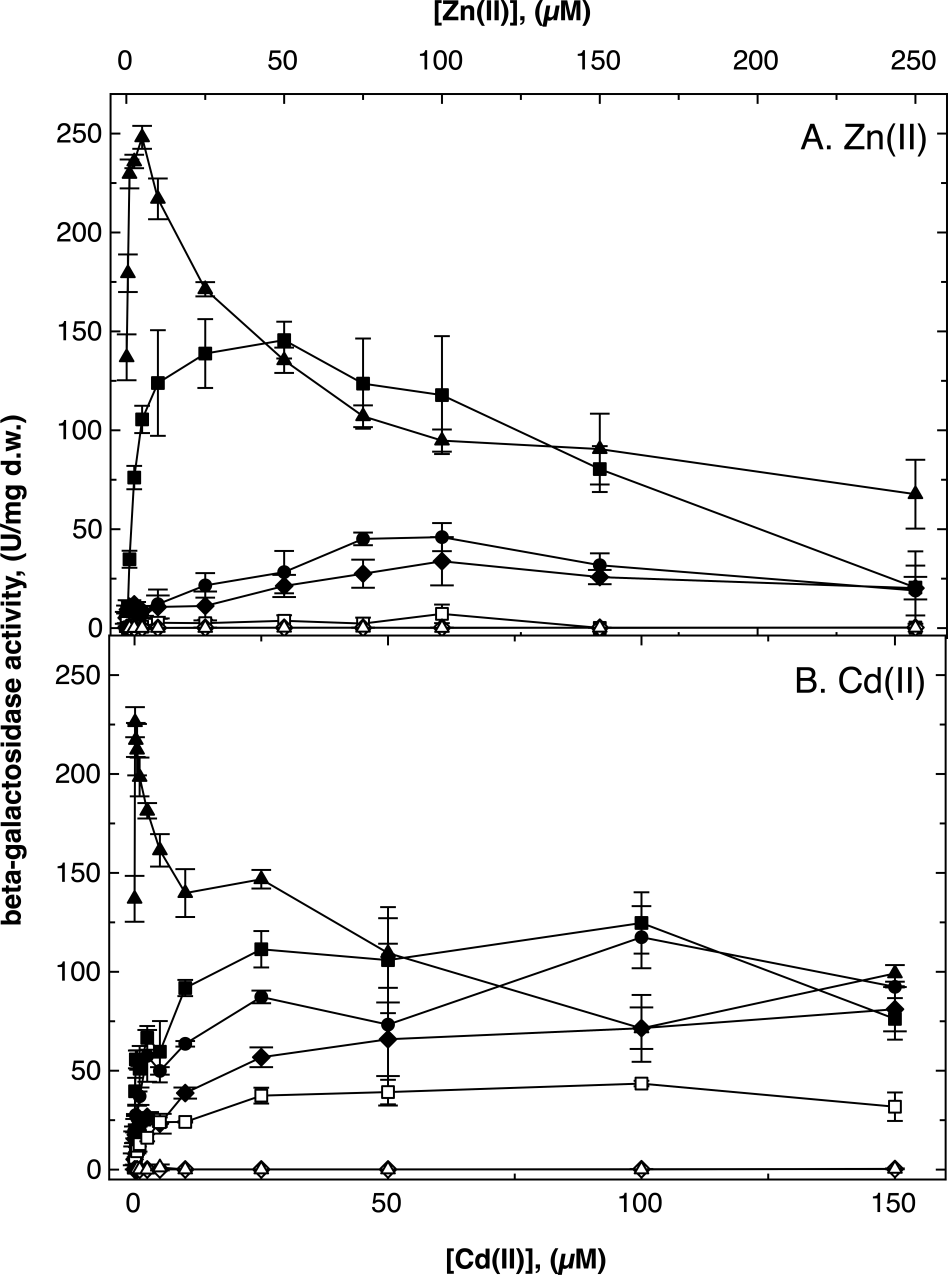
Regulation of *cdfX* expression as determined with a Φ(*cdfX-lacZ*) reporter gene fusion. The beta-galactosidase activity of a Φ(*cdfX::lacZ*) transcriptional fusion in response to increasing zinc (Panel A) or cadmium (Panel B) concentrations was determined in strain AE104 (closed circles), ∆*zntA* (closed squares), ∆*cadA* (closed diamonds), ∆*zntA ∆cadA* (closed triangles), and additional ∆*zntR* deletions in the strains ∆*zntA* (open squares), ∆*cadA* (open diamonds), and ∆*zntA ∆cadA* (open triangles). Strains cultivated in TMM, *n* = 3, deviations shown.

A Φ(*cdfX-lacZ*) transcription fusion that did not interrupt the *cdfX* gene was upregulated by increasing intracellular zinc and cadmium concentrations in the parent AE104 (Fig. 4). Upregulation increased when the major zinc efflux pump, ZntA, but not its paralog CadA, was absent. A ∆*zntA ∆cadA* double deletion strain showed strong upregulation of *cdfX* expression at low zinc and cadmium concentrations, with maxima at 5 µM Zn(II) and 100 nM Cd(II), respectively, indicating that expression of *cdfX* is controlled in response to zinc and cadmium levels. Because upregulation was higher in cells without the two metal-exporting P_IB2_-type ATPases, this suggests that the signal being sensed seems to be cytoplasmic Zn(II) and Cd(II) ions. Since a ∆*cadA* deletion also removes the *cadRp* promoter region, Zn- and Cd-regulated expression could be associated with the *cdfXp* promoter directly upstream of *cdfX* (Fig. S1). In electrophoretic mobility shift assay (EMSA) experiments performed previously (20), ZntR bound to this *cdfXp* promoter (Fig. S2). For a deeper analysis, the dependence of the retardation on the ZntR:promoter ratio was quantified (Fig. S3). ZntR bound to *zntRp* following a sigmoidal function with a turn-point of the function at a ZntR:promoter ratio between 25 and 50. This may indicate cooperative binding of ZntR to its own promoter with 50% occupation *in vitro* at a ZntR concentration of about 0.5 µM. In contrast, binding to *cdfXp* occurred step-wisely with 50% occupation at much higher concentrations of about 10 µM *in vitro*. This was reminiscent to the binding of ZntR to *zntAp*, the promoter of the gene for the zinc-exporting P-type ATPase (20). These data indicate that ZntR is the main regulator of *cdfX* expression.

Consequently, deletion of *zntR* completely abolished the zinc-specific upregulation of Φ(*cdfX-lacZ*) expression (Fig. 4). The ∆*zntR ∆zntA* double-null mutant, but not the ∆*zntR ∆cadA* or the ∆*zntR ∆cadA ∆zntA* mutant, displayed a residual Cd-dependent upregulation of expression of the *cdfX-lacZ* fusion. Since all ∆*cadA* mutants also carried a deletion in the *cadRp* promoter region, this indicated a residual, cadmium-dependent expression of *cdfX* from *cadRp* (Fig. S1). As expected, when Zn- and Cd-dependent expression of a Φ(*cadR-lacZ*) reporter gene fusion was analyzed, no expression occurred in the ∆*cadA* and ∆*cadA ∆zntA* background (Fig. S4). In all other strains, expression decreased with increasing metal concentrations, which could be an effect of metal toxicity. Alternatively, synthesis of CadR, which may be a repressor (20), was downregulated, so that its target genes were derepressed at high metal concentrations.

Cobalt was not an inducer of Φ(*cdfX-lacZ*) expression in AE104 and most other strains, including ∆*cadA* (Fig. S5), but a low level of upregulation occurred in the ∆*zntA* background. The ∆*zntA ∆cadA* mutant expressed the reporter fusion at a high constitutive level (Fig. S5) while Zn and Cd mediated a concentration-dependent downregulation of reporter expression in this mutant (Fig. 4). The constitutively high expression level in the absence of metals or the presence of cobalt was completely abolished by an additional ∆*zntR* deletion. This demonstrated ZntR-dependent high-level expression of *cdfX* from *cdfXp* when metal concentrations are low in cells without CadA and ZntA, and this expression was downregulated by increasing Zn and Cd concentrations, but was unresponsive to Co increasing concentrations, probably due to the presence of the cobalt exporter DmeF (Fig. S5).

### ZntR and CadR affect metal resistance

Metal resistance of the parental strain AE104 and its mutants was determined in a dose–response analysis in liquid TMM. The lower IC_50_ of the parent compared to the published value was due to a higher zinc content in the growth medium during the previous study (19). As published here and elsewhere (18), deletion of *zntA*, but not that of *cadA*, decreased zinc resistance by half, while it decreased to 5% when both pumps were absent. Determination of the MIC on solid medium revealed a similar effect (Table S1). Both pumps contributed synergistically to cadmium resistance in liquid medium but substituted for each other with respect to cadmium resistance on agar plates, while CadA was only a backup system for zinc resistance in the presence of ZntA. Cobalt resistance decreased in the ∆*zntA* but not the *∆cadA* single or the double mutant, so the absence of ZntA caused a CadA-dependent loss of Co tolerance. This effect was not visible on solid medium and was likely the result of a disturbance in the linked zinc and cobalt homeostasis (9).

Deletion of *zntR*, or *cadR*, or of both genes, decreased the IC_50_ for zinc, with the value of the double deletion strain lying between that of the single deletion mutants (Table 3). On solid medium, deletion of *cadR* had no effect on zinc resistance. Deletion of *zntR*, as well as the MIC of the double null mutant, resulted in a very similar phenotype to that of the ∆*zntR* mutant. Thus, although both regulators contributed to zinc resistance, CadR only showed an effect in liquid medium. Removal of the *zntR* gene by disruption or marker-free deletion in the ∆*cadA* strain decreased the IC_50_ of the resulting strain to that of the ∆*zntA ∆cadA* double mutant, which highlighted again the essential role of ZntR in expression of *zntA*. Without ZntR, the IC_50_ of the double mutant ∆*zntA ∆cadA* decreased, which agreed with the zinc resistance values of the ∆e4 ∆*cdfX* mutant compared to the ∆e4 parent (Fig. 2) and with the contribution of ZntR to expression of *cdfX* (Fig. 4). This effect was also evident on agar plates (Table S1). Alternatively, it could be explained by the ZntR-dependent expression of *cdfX* or a ZntR-independent residual expression of *zntA*. Because ZntR was essential for *zntA* expression (20), the ZntR-dependent expression of *cdfX* is currently the more plausible explanation.

**TABLE 3:** Deletions of the genes for the regulators decrease metal resistance^*a*^

Bacterial strain	IC_50_ values (µM)			
	Zn	Cd	Co	EDTA
AE104	115 ± 16	136 ± 14	154 ± 28	1409 ± 135
*△zntA*	53.0 ± 5.2	37.7 ± 8.0	77.4 ± 10.9	1574 ± 177
*△cadA*	112 ± 9	87.5 ± 5.2	151 ± 20	1579 ± 101
*△zntA △cadA*	5.6 ± 0.1	0.1 ± 0.0	194 ± 15	1740 ± 62
*△zntR*	52.8 ± 2.6	59.0 ± 3.6	185 ± 4	1338 ± 118
*△cadR*	73.1 ± 1.5	206 ± 14	151 ± 6	1265 ± 16
*△zntR △cadR*	63.9 ± 0.5	176 ± 13	193 ± 3	1445 ± 133
*△zntA zntR::dis*	23.1 ± 0.9	36.6 ± 4.0	64.2 ± 5.3	1521 ± 35
*△cadA zntR::dis*	5.5 ± 0.0	0.1 ± 0.0	114 ± 15	1444 ± 34
*△zntA △cadA zntR::dis*	2.5 ± 0.0	0.1 ± 0.0	137 ± 5	1446 ± 36
*△zntA △zntR*	55.7 ± 2.8	44.2 ± 2.4	83.2 ± 0.9	1501 ± 53
*△cadA △zntR*	5.9 ± 0.1	0.1 ± 0.0	202 ± 11	1598 ± 46
*△zntA △cadA △zntR*	3.7 ± 0.0	0.1 ± 0.0	195 ± 8	1601 ± 51

^*a*^ Dose response curves were performed in TMM with an ambient zinc concentration (200 nM) with increasing metal or EDTA concentrations for 25 h and the IC_50_ was calculated. Values for ∆*zntA, ∆cadA*, and ∆*zntA ∆cadA* obtained with a TMM with a higher zinc concentration were previously published (19). This different medium resulted in a higher zinc resistance of the parent. Values previously published for ∆*zntR* and ∆*cadR* were from cells with standard TMM (160 nM Zn) and comparable (20). The published experiments were repeated here and given for comparison.

Deletion of *zntR* decreased the IC_50_ for cadmium while that of *cadR* increased it (Table 3), with the Cd(II) resistance of the double mutant not being different from that of the *cadR* mutant. Again, as previously noted (20), CadR but not ZntR seems to repress the expression of the genes responsible for conferring cadmium resistance. While deletion of *zntR* in a ∆*zntA* mutant did not affect the IC_50_ for Cd(II) when compared to the ∆*zntA* single mutant, resistance was reduced to IC_50_ = 100 nM, similar to the effect of the ∆*zntA* deletion being introduced into ∆*cadA* deletion, when the ∆*zntR* allele was introduced into both ∆*cadA* mutant and the ∆*zntA* ∆*cadA* strain. A similar result was obtained on solid medium (Table S1). This indicated again the essential contribution of ZntR to *zntA* expression, which was important for cadmium resistance in the absence of CadA. There was no effect of the deletion of these regulatory genes on cobalt or EDTA resistance (Table 3).

### Regulation of *zntR* expression

**Fig 5:**
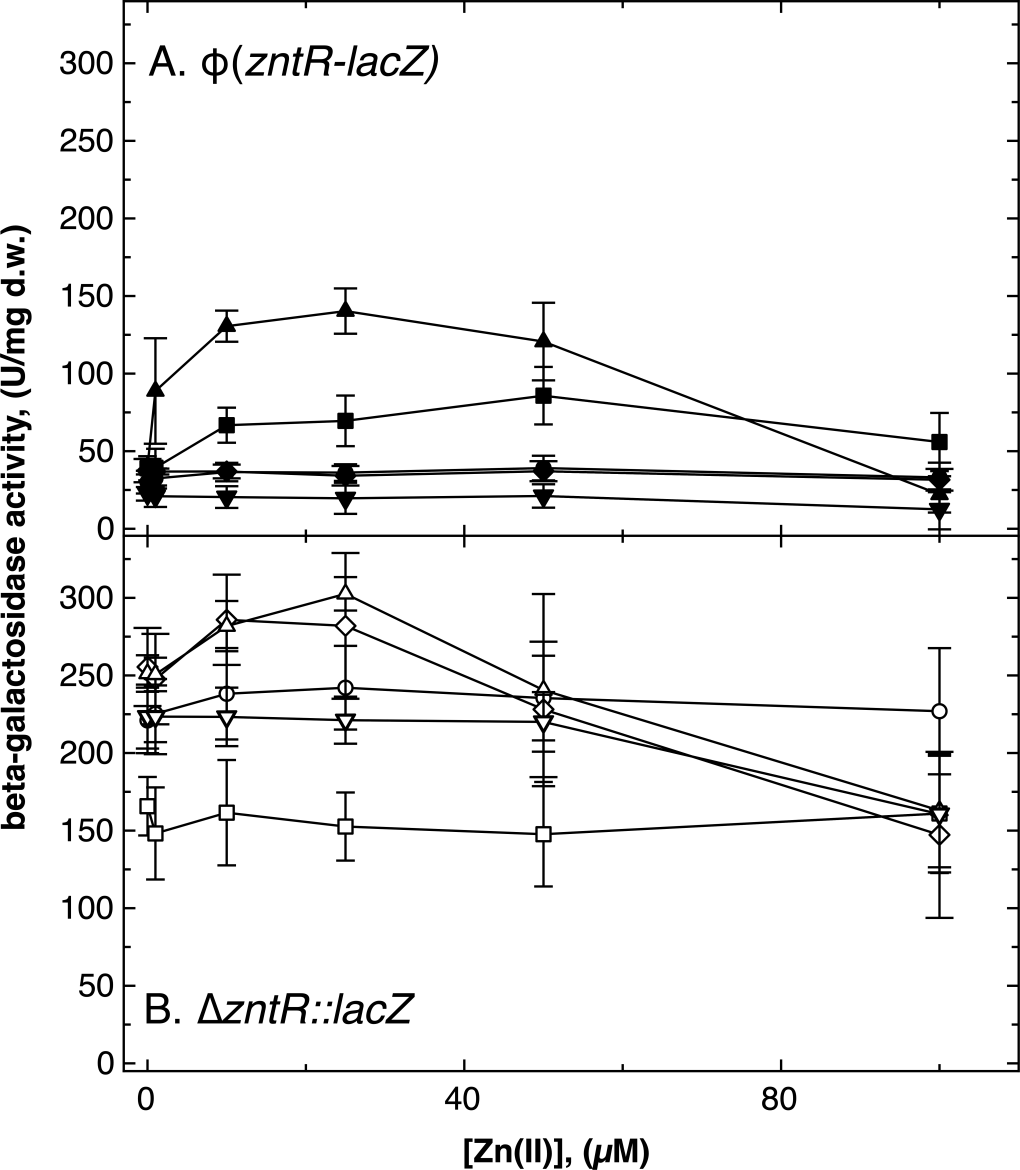
Regulation of *zntR* by zinc as determined with a *zntR-lacZ* reporter gene fusions. The beta-galactosidase enzyme activity of a *zntR-lacZ* transcriptional fusion in response to increasing zinc concentrations was determined in strain AE104 (circles), ∆*zntA* (squares), ∆*cadA* (diamonds), ∆*zntA ∆cadA* (triangles), and ∆*cadR* (inverted triangles) deletion mutants. Panel A shows ϕ(*zntR::lacZ*) fusion that did not mutate *zntR* (closed symbols) and Panel B ∆*zntR::lacZ* fusions that interrupted *zntR* (open symbols). Strains cultivated in TMM, *n* ≥ 3, deviations shown.

To understand what regulated synthesis of the regulator ZntR, transcriptional reporter gene fusions of *zntR* were constructed generating, on the one hand, strain Φ(*zntR-lacZ*) that retained a functional *zntR* gene, and on the other hand, a strain ∆*zntA::lacZ* in which *zntR* was insertionally inactivated using the *lacZ* gene. This created cells that could autoregulate *zntR* expression or not. No upregulation of expression of the *zntR-lacZ* fusion in strain Φ(*zntR-lacZ*) could be measured in the parent AE104, in the ∆*cadA*, or in the ∆*cadR* background but *zntR* was expressed constitutively. However, in the ∆*zntA* strain, and even more so in the ∆*zntA ∆cadA* strains, Φ(*zntR-lacZ*) was upregulated in the presence of exogenous zinc (Fig. 5A). Expression of *zntR* was thus responsive to cytoplasmic zinc concentrations.

In the ∆*zntR::lacZ* strain with a disrupted *zntR* gene, expression was on a higher basic level compared to that of the Φ(*zntR-lacZ*) strains (Fig. 5B). Absence or presence of CadR made no difference to *zntR* expression levels and no regulation by zinc was measurable in either the parental or *∆cadR* background. There was also no zinc-dependent regulation in the ∆*zntA* background and expression was at a lower basic activity level. On the other hand, *∆zntR::lacZ* was regulated by the zinc concentration in the ∆*cadA* and ∆*zntA ∆cadA* strains, and that in a similar fashion. This indicated that in the ∆*zntA* mutant, the presence of the complete *cad* determinant with *cadA* and *cadR* expressed from an overlapping promoter region (Fig. S1) prevented *zntR*-independent expression of *zntR*. The ZntR- and CadR-dependent regulatory pathways seem to be interwoven. Regulation of *zntR*-expression followed a pattern similar to that of Zn in the presence of Cd (Fig. S6) or Co (Fig. S7) with only minor differences.

## DISCUSSION

### Zinc starvation

A schematic representation (Fig. 1) illustrates the findings of this study and integrates them into the model for zinc homeostasis in *C. metallidurans*. The bacterium accumulates and maintains about 70,000–80,000 Zn ions per cell (10, 13, 42, 43). Under conditions of low zinc availability, the Zur regulator, which has an ortholog in a multitude of other bacteria (44–50), derepresses expression of the *zupT* gene and also two other gene regions (43, 51, 52). One contains *zur* and two genes for proteins of the COG0523-family of GTPases (53, 54), *cobW2* and *cobW3* (51, 52), the second *cobW1* and paralogs for Zn-dependent or Zn-salvage proteins, for instance the metal promiscuous GTP-cyclohydrolase FolE_IB2 that substitutes its zinc-dependent paralog FolE_IA under conditions of zinc starvation (55–59). At least one of these enzymes is essential because its GTP cyclohydrolase activity is responsible for catalyzing the initial step of folate biosynthesis (53). Since formyl-tetrahydrofolate is essential for GTP biosynthesis and GTP for folate biosynthesis, the metal-dependent FolE-catalysis represents an “Achilles heel” in cellular metabolism (60, 61). Together with ZupT, CobW2 and CobW3 control zinc homeostasis by directing the flow equilibrium of zinc, which results from the uptake and efflux reactions. Together with the cobalt efflux system DmeF, this trio also controls an increased accumulation of Co(II) ions under zinc-starvation conditions, which may fill up the zinc pool with cobalt ions to a summary Co + Zn content of 83,000 metal ions. This reaction protects the zinc pool against cadmium toxicity and may provide Co(II) to metal-promiscuous enzymes such as FolE_IB1 and FolE_IB2 (9, 55). The proteins CobW1, FolE_IB, Zur, and ZupT also have homologs in many other bacteria (45–50, 54, 62–64). The ability to survive zinc starvation conditions seems to be a fundamental ability of most bacteria. In *C. metallidurans*, it is the fundament for the additional acquisition of the ability to survive metal stress.

### Ambient and slightly elevated zinc concentrations

At external zinc concentrations above 200 nM, representing more than 80,000 Zn ions per cell, expression of the *zntA* gene, encoding the central zinc and cadmium-exporting P_IB2_-type ATPase ZntA, is upregulated (18, 20). The MerR-type regulator ZntR is responsible for upregulation of *zntA* (20). The *zntR* gene is also located on the chromosome but without genes involved in metal resistance in its vicinity. ZntR and ZntA also have orthologs in many bacteria, including *Escherichia coli* (24–26, 65, 66). ZntR-mediated upregulation of *zntA* is zinc- or cadmium-dependent and the efflux pumps transport both cations with equal efficiency (19) such that a basic resistance level to cadmium and moderate concentration of zinc is mediated by ZntR and ZntA.

Cd(II) is present in zinc-rich minerals with a few percent of the zinc content (67). Similar to Au, Ag, and Hg, Cd is a transition metal with high mobility despite a comparable low content in the earth’s crust, whereas Zn, similar to for example Ni, Cu, and Co, is a metal with high availability (39). This means that *C. metallidurans* likely encounters cadmium in its metal-rich environment, especially those rich in zinc.

Another P_IB2_-type ATPase, CadA, is a known backup system for ZntA (18). Both proteins transport Zn(II) and Cd(II) with similar kinetic parameters *in vitro* (19), however, they contain different metal-binding motifs (40). ZntA has a large His-rich stretch of 20 amino acids at its N-terminal region, followed by a triple-Cys site and a heavy-metal associated (HMA) motif, termed type cd00371. CadA, in contrast, starts with four HMA motifs at its N-terminus (40). The metal-binding motifs of P_IB_-type ATPases are involved in acquisition of their substrates and are also required for flux control of the transport activity (68–71). Consequently, CadA and ZntA may have different functions *in vivo*. Regulation of *cadA* expression is by the MerR-type regulator CadR (20), which is encoded upstream of *cadA* but on the other DNA strand, so that expression of both genes is initiated from a common promoter-operator region (Fig. S1). The physiological data indicate that CadR may be a repressor (20), which is an unusual feature for a MerR-type metal regulator (26). Unfortunately, purification of CadR has not been successful therefore biochemical analysis of the protein has not been possible. Nevertheless, the experimental evidence available suggests that CadR derepresses *cadA* expression under conditions of high intracellular cadmium concentrations. CadR-CadA could be involved in resolving an imbalance in cellular zinc and cadmium concentrations, with cadmium disturbing the flow equilibrium of zinc by competition with Zn(II) for export by ZntA.

These previously published data (20) explain what happens in *C. metallidurans* when the cadmium concentration is too high for ZntA to deal with alone, even after ZntR-dependent upregulation. The data did not explain, however, what happens when an imbalance in zinc and cadmium concentrations results in competition between Zn(II) and Cd(II) for ZntA, which would disturb the flow equilibrium causing an increased zinc concentration. Searching for a zinc efflux system responsible for a residual export activity in pulse-chase experiments with the ∆4e mutant (∆*zntA ∆cadA ∆dmeF ∆fieF*) of *C. metallidurans* identified CdfX, which is a candidate to fulfill this function. This *cdfX* gene is located downstream of *cadR* with two other genes on opposite DNA strands in-between, which are both not expressed in unchallenged *C. metallidurans* cells (Fig. S1). The *cad* region is located on genomic island CMGI_1 of the chromosome and thus was presumably obtained by horizontal gene transfer (31).

The *cdfX* gene is expressed under zinc- and cadmium-dependent control of ZntR (Fig. 4) and encodes an unusual CDF protein, which lacks the cytoplasmic domain present in most CDF proteins, such as DmeF, FieF (Fig. S1) and Yiip (35–38). CdfX is reminiscent of the P_IB_-type ATPases, with this region in these proteins being involved in substrate acquisition, flux control of the transport activity and dimerization. The *cdfXp* EMSA (Fig. S2 and S3) and *cdfX-lacZ* reporter data (Fig. 4) compared to those published for *zntA* indicate that *cdfX* and *zntA* are expressed under zinc control in a similar way with an optimum at 100 µM Zn(II) in the medium (18, 20). Expression of *cdfX* was upregulated at much lower concentrations in the absence of ZntA and even lower concentrations when both, ZntA and CadA, were absent. This would mean that the P_IB2_-type ATPases ZntA and CadA might be more efficient zinc exporters than CdfX. Moreover, the contribution of the CDF protein CzcD to cadmium resistance is much lower than that of ZntA or CadA (19), indicating that CDF proteins are mainly efflux systems for transition metal cations of the first row of the periodic systems of the elements. CdfX might be an additional export pathway for zinc ions not exported with sufficient rate by ZntA. This would mean that during its evolution *C. metallidurans* has acquired by horizontal gene transfer this *cad* region to manage imbalanced zinc and cadmium levels by controlled expression of *cdfX* or *cadA*, respectively.

### Zinc resistance

All the factors discussed up to now mediate a degree of metal resistance and zinc-starvation management in the plasmid-free strain *C. metallidurans* AE104, which is on a similar level as that of other bacteria, for instance, *E. coli* (6, 19). High levels of metal resistance are mediated by plasmid-encoded proteins (6, 72, 73). The *czc* determinant on plasmid pMOL30 encodes the transenvelope system CzcCBA, the CDF protein CzcD, the two-component regulatory system CzcRS and three periplasmic proteins CzcI, CzcE, and CzcJ (74–76). CzcCBA exports Zn(II), Co(II), and Cd(II) from the periplasm to the outside, antagonizing import of these metals by porins. This results in a decrease in the flow equilibrium of zinc because the substrate concentration for the metal import systems of the inner membrane is diminished (23). CzcI and its chromid-encoded paralog quench the activity of CzcCBA to decrease efflux of cobalt, which may be needed to substitute an imbalanced zinc pool in the cytoplasm (20). The function of CzcJ is still unknown and CzcE interacts with CzcD and CzcRS (74).

At very high zinc concentrations, the response regulator CzcR activates *czc* expression from another promoter and that of *czcP* (19, 74). CzcP is a P_IB4_-type ATPase that transports zinc at a higher rate than ZntA but is not able to take over the functions of ZntA or CadA (19, 77). CzcP seems to be able to catalyze a rapid export of more loosely bound cytoplasmic zinc for subsequent further export by CzcCBA.

### Conclusion

Zinc homeostasis of *C. metallidurans* could be understood as being organized in layers (Fig. 1). All these functions enable *C. metallidurans* to survive zinc concentrations from about 30 nM to 10 mM. At the lower end are zinc concentrations that barely allow the allocation of sufficient zinc to zinc-dependent proteins such as RpoC. Components of the zinc starvation layers are Zur, ZupT, CobW2, and CobW3. For extreme zinc starvation conditions, ZagA-ZigA-CobW1-type GTPases use GTP-bound energy to deliver zinc to important zinc-dependent proteins or prevent activation of metal-promiscuous zinc-salvage enzymes. On the other hand, if zinc concentrations approach the maximum solubility of Zn(OH)_2_ at neutral pH values, which is about 4.5 mM (67), transenvelope efflux systems and two-component regulators come into play, which remove periplasmic zinc cations before they reach the metal import systems. In parallel, additional efflux systems of the inner membrane are upregulated. Zinc homeostasis at ambient zinc concentrations is governed by the flow equilibrium of uptake and efflux reactions. In the cases of Zn(II) and Cd(II), efflux is mediated by ZntA. In the case of an imbalance in zinc and cadmium content, CadA removes Cd(II), while CdfX removes Zn(II). This protects the flow equilibrium of zinc when ZntA is overwhelmed by the competition between Zn(II) and Cd(II) for export by this protein.

## MATERIALS AND METHODS

### Bacterial strains and growth conditions

Strains used for experiments were derivatives of the plasmid-free derivative AE104 of *C. metallidurans* CH34 (6) and are listed in Table S2. Tris-buffered mineral salts medium (6) containing 2 g sodium gluconate/l (TMM) was used to cultivate these strains aerobically with shaking at 30°C. TMM with different zinc concentrations was used as published (9), ambient zinc TMM (aZn) with 200 nM Zn(II), standard TMM with 160 ± 36 nM Zn(II) (TMM), moderate zinc-containing medium (mZn) with 64 ± 9 aM Zn(II) or low zinc, low magnesium medium with 40 nM Zn and 100 µM Mg(II) instead of 1 mM (lZn-lMg). Solid Tris-buffered media contained 20 g agar/L. Strains were routinely transferred to fresh TMM plates every 2 weeks and taken from the −80°C stock culture twice a year.

### Genetics, measurements, determination of resistance, and reporter genes

Dose–response growth curves in 96-well plates, construction of reporter genes, ß-galactosidase assays, gene deletions, disruptions, construction of reporter operon fusions, and other genetic methods were performed as published recently (9, 55). For pulse-chase experiments with radioactive ^65^Zn, experiments with stable ^67^Zn and inductively-coupled plasma mass spectrometry (ICP-MS) please refer to the recently published detailed description (23).

### Electrophoretic mobility shift assay

Gel retardation assays were performed as published (75) with modifications. PCR products together with purified recombinant proteins were used. Approximately 0.4 pmol of the *cdfXp* and *zntRp* promoter fragment and various concentrations of ZntR were used for each assay. The DNA-protein complex was formed at 30°C for 40 min in 30 µL binding buffer [50 mM KCl, 20 mM Tris-HCl (pH 7.8), 5% glycerol, and 1 mM DTT]. The reactions were cooled on ice and applied to a 10% PAA gel with a running buffer containing 1× native buffer (100 mM Tris, 100 mM glycine). The gel was run at 4°C and 60 V for 1–2 h, stained with HD green plus (Intas, Göttingen, Germany) or silver, dried and densitometrically scanned using ImageJ (78).

### Statistics

Students’ *t* test was used but in most cases the distance (*D*) value, *D*, has been used several times previously for such analyses (12, 79, 80). It is a simple, more useful value than Student’s *t* test because non-intersecting deviation bars of two values (*D* > 1) for three repeats always mean a statistically relevant (≥95%) difference provided the deviations are within a similar range. At *n* = 4, significance is ≥97.5%, at *n* = 5 ≥ 99% (significant) and at *n* = 8 ≥ 99.9% (highly significant).

## SUPPLEMENTAL MATERIAL

Supplemental figures and tables jb.00299-24-s0001.pdf Fig. S1 to S7; Tables S1 and S2.

ASM does not own the copyrights to Supplemental Material that may be linked to, or accessed through, an article. The authors have granted ASM a non-exclusive, world-wide license to publish the Supplemental Material files. Please contact the corresponding author directly for reuse.

## References

[1] Reith F, Rogers SL, McPhail DC, Webb D. 2006. Biomineralization of gold: biofilms on bacterioform gold. Science 313:233–236. doi:10.1126/science.1125878 16840703

[2] Diels L, Mergeay M. 1990. DNA probe-mediated detection of resistant bacteria from soils highly polluted by heavy metals. Appl Environ Microbiol 56:1485–1491. doi:10.1128/aem.56.5.1485-1491.1990 16348196 PMC184435

[3] Mergeay M, Van Houdt R. 2021. Cupriavidus metallidurans CH34, a historical perspective on its discovery, characterization and metal resistance. FEMS Microbiol Ecol 97:fiaa247. doi:10.1093/femsec/fiaa247 33270823

[4] Nies DH. 2016. The biological chemistry of the transition metal “transportome” of Cupriavidus metallidurans. Metallomics 8:481–507. doi:10.1039/c5mt00320b 27065183

[5] Janssen PJ, Van Houdt R, Moors H, Monsieurs P, Morin N, Michaux A, Benotmane MA, Leys N, Vallaeys T, Lapidus A, Monchy S, Médigue C, Taghavi S, McCorkle S, Dunn J, van der Lelie D, Mergeay M. 2010. The complete genome sequence of Cupriavidus metallidurans strain CH34, a master survivalist in harsh and anthropogenic environments. PLoS One 5:e10433. doi:10.1371/journal.pone.0010433 20463976 PMC2864759

[6] Mergeay M, Nies D, Schlegel HG, Gerits J, Charles P, Van Gijsegem F. 1985. Alcaligenes eutrophus CH34 is a facultative chemolithotroph with plasmid-bound resistance to heavy metals. J Bacteriol 162:328–334. doi:10.1128/jb.162.1.328-334.1985 3884593 PMC218993

[7] Liesegang H, Lemke K, Siddiqui RA, Schlegel H-G. 1993. Characterization of the inducible nickel and cobalt resistance determinant cnr from pMOL28 of Alcaligenes eutrophus CH34. J Bacteriol 175:767–778. doi:10.1128/jb.175.3.767-778.1993 8380802 PMC196216

[8] Nies DH. 2013. RND-efflux pumps for metal cations, p 79–122. In Yu EW, Zhang Q, Brown MH (ed), Microbial efflux pumps: current research. Caister Academic Press, Norfolk, UK.

[9] Galea D, Herzberg M, Nies DH. 2024. The metal-binding GTPases CobW2 and CobW3 are at the crossroads of zinc and cobalt homeostasis in Cupriavidus metallidurans. J Bacteriol 206:e0022624. doi:10.1128/jb.00226-24 39041725 PMC11340326

[10] Herzberg M, Dobritzsch D, Helm S, Baginsky S, Nies DH. 2014. The zinc repository of Cupriavidus metallidurans. Metallomics 6:2157–2165. doi:10.1039/C4MT00171K 25315396

[11] Herzberg M, Bauer L, Kirsten A, Nies DH. 2016. Interplay between seven secondary metal uptake systems is required for full metal resistance of Cupriavidus metallidurans. Metallomics 8:313–326. doi:10.1039/c5mt00295h 26979555

[12] Große C, Herzberg M, Schüttau M, Wiesemann N, Hause G, Nies DH. 2016. Characterization of the Δ7 mutant of Cupriavidus metallidurans with deletions of seven secondary metal uptake systems. mSystems 1:e00004-16. doi:10.1128/mSystems.00004-16 27822513 PMC5069749

[13] Kirsten A, Herzberg M, Voigt A, Seravalli J, Grass G, Scherer J, Nies DH. 2011. Contributions of five secondary metal uptake systems to metal homeostasis of Cupriavidus metallidurans CH34. J Bacteriol 193:4652–4663. doi:10.1128/JB.05293-11 21742896 PMC3165648

[14] Fagan MJ, Saier MH Jr. 1994. P-type ATPases of eukaryotes and bacteria: sequence analyses and construction of phylogenetic trees. J Mol Evol 38:57–99. doi:10.1007/BF00175496 8151716

[15] Paulsen IT, Saier MH Jr. 1997. A novel family of ubiquitous heavy metal ion transport proteins. J Membr Biol 156:99–103. doi:10.1007/s002329900192 9075641

[16] Munkelt D, Grass G, Nies DH. 2004. The chromosomally encoded cation diffusion facilitator proteins DmeF and FieF from Wautersia metallidurans CH34 are transporters of broad metal specificity. J Bacteriol 186:8036–8043. doi:10.1128/JB.186.23.8036-8043.2004 15547276 PMC529076

[17] Grass G, Otto M, Fricke B, Haney CJ, Rensing C, Nies DH, Munkelt D. 2005. FieF (YiiP) from Escherichia coli mediates decreased cellular accumulation of iron and relieves iron stress. Arch Microbiol 183:9–18. doi:10.1007/s00203-004-0739-4 15549269

[18] Legatzki A, Grass G, Anton A, Rensing C, Nies DH. 2003. Interplay of the Czc system and two P-type ATPases in conferring metal resistance to Ralstonia metallidurans. J Bacteriol 185:4354–4361. doi:10.1128/JB.185.15.4354-4361.2003 12867443 PMC165768

[19] Scherer J, Nies DH. 2009. CzcP is a novel efflux system contributing to transition metal resistance in Cupriavidus metallidurans CH34. Mol Microbiol 73:601–621. doi:10.1111/j.1365-2958.2009.06792.x 19602147

[20] Schulz V, Schmidt-Vogler C, Strohmeyer P, Weber S, Kleemann D, Nies DH, Herzberg M. 2021. Behind the shield of Czc: ZntR controls expression of the gene for the zinc-exporting P-type ATPase ZntA in Cupriavidus metallidurans J Bacteriol 203:e00052-21. doi:10.1128/JB.00052-21 33685972 PMC8117531

[21] Taghavi S, Lesaulnier C, Monchy S, Wattiez R, Mergeay M, van der Lelie D. 2009. Lead(II) resistance in Cupriavidus metallidurans CH34: interplay between plasmid and chromosomally-located functions. Antonie Van Leeuwenhoek 96:171–182. doi:10.1007/s10482-008-9289-0 18953667

[22] Borremans B, Hobman JL, Provoost A, Brown NL, van Der Lelie D. 2001. Cloning and functional analysis of the pbr lead resistance determinant of Ralstonia metallidurans CH34. J Bacteriol 183:5651–5658. doi:10.1128/JB.183.19.5651-5658.2001 11544228 PMC95457

[23] Nies DH, Schleuder G, Galea D, Herzberg M. 2024. A flow equilibrium of zinc in cells of Cupriavidus metallidurans J Bacteriol 206:e0008024. doi:10.1128/jb.00080-24 38661374 PMC11112998

[24] Brocklehurst KR, Hobman JL, Lawley B, Blank L, Marshall SJ, Brown NL, Morby AP. 1999. ZntR is a Zn(II)-responsive MerR-like transcriptional regulator of zntA in Escherichia coli. Mol Microbiol 31:893–902. doi:10.1046/j.1365-2958.1999.01229.x 10048032

[25] Outten CE, O’Halloran TV. 2001. Femtomolar sensitivity of metalloregulatory proteins controlling zinc homeostasis. Science 292:2488–2492. doi:10.1126/science.1060331 11397910

[26] Outten CE, Outten FW, O’Halloran TV. 1999. DNA distortion mechanism for transcriptional activation by ZntR, a Zn(II)-responsive MerR homologue in Escherichia coli. J Biol Chem 274:37517–37524. doi:10.1074/jbc.274.53.37517 10608803

[27] Tulin G, Figueroa NR, Checa SK, Soncini FC. 2024. The multifarious MerR family of transcriptional regulators. Mol Microbiol 121:230–242. doi:10.1111/mmi.15212 38105009

[28] Hobman JL, Yamamoto K, Oshima T. 2007. Transcriptomic responses of bacterial cells to sublethal metal ion stress, p 73–116. In Nies DH, Silver S (ed), Molecular microbiology of heavy metals. Vol. 6. Springer-Verlag, Berlin.

[29] Helmann JD, Soonsange S, Gabriel S. 2007. Metallalloregulators: arbiters of metal sufficiency, p 37–71. In Nies DH, Silver S (ed), Molecular microbiology of heavy metals. Vol. 6. Springer-Verlag, Berlin.

[30] Van Houdt R, Monsieurs P, Mijnendonckx K, Provoost A, Janssen A, Mergeay M, Leys N. 2012. Variation in genomic islands contribute to genome plasticity in Cupriavidus metallidurans. BMC Genomics 13:111. doi:10.1186/1471-2164-13-111 22443515 PMC3384475

[31] Van Houdt R, Monchy S, Leys N, Mergeay M. 2009. New mobile genetic elements in Cupriavidus metallidurans CH34, their possible roles and occurrence in other bacteria. Antonie Van Leeuwenhoek 96:205–226. doi:10.1007/s10482-009-9345-4 19390985

[32] Große C, Grau J, Große I, Nies DH. 2022. Importance of RpoD- and Non-RpoD-dependent expression of horizontally acquired genes in Cupriavidus metallidurans. Microbiol Spectr 10:e0012122. doi:10.1128/spectrum.00121-22 35311568 PMC9045368

[33] Große C, Kohl TA, Niemann S, Herzberg M, Nies DH. 2022. Loss of mobile genomic Islands in metal-resistant, hydrogen-oxidizing Cupriavidus metallidurans. Appl Environ Microbiol 88:e0204821. doi:10.1128/AEM.02048-21 34910578 PMC8862790

[34] Varadi M, Anyango S, Deshpande M, Nair S, Natassia C, Yordanova G, Yuan D, Stroe O, Wood G, Laydon A, et al.. 2022. AlphaFold Protein Structure Database: massively expanding the structural coverage of protein-sequence space with high-accuracy models. Nucleic Acids Res 50:D439–D444. doi:10.1093/nar/gkab1061 34791371 PMC8728224

[35] Chao Y, Fu D. 2004. Thermodynamic studies of the mechanism of metal binding to the Escherichia coli zinc transporter YiiP. J Biol Chem 279:17173–17180. doi:10.1074/jbc.M400208200 14960568

[36] Lu M, Chai J, Fu D. 2009. Structural basis for autoregulation of the zinc transporter YiiP. Nat Struct Mol Biol 16:1063–1067. doi:10.1038/nsmb.1662 19749753 PMC2758918

[37] Lu M, Fu D. 2007. Structure of the zinc transporter YiiP. Science 317:1746–1748. doi:10.1126/science.1143748 17717154

[38] Wei YN, Fu D. 2005. Selective metal binding to a membrane-embedded aspartate in the Escherichia coli metal transporter YiiP (FieF). J Biol Chem 280:33716–33724. doi:10.1074/jbc.M506107200 16049012

[39] Nies DH. 2022. Chemical constraints for transition metal cation allocation, p 21–52. In Hurst CJ (ed), Microbial metabolism of metals and metalloids. Vol. 10. Springer, Heidelberg.

[40] Nies DH. 2022. How is a zinc ion correctly allocated to a zinc-dependent protein?, p 579–659. In Hurst CJ (ed), Microbial metabolism of metals and metalloids. Vol. 10. Springer, Heidelberg.

[41] Nies DH, Silver S. 1989. Metal ion uptake by a plasmid-free metal-sensitive Alcaligenes eutrophus strain. J Bacteriol 171:4073–4075. doi:10.1128/jb.171.7.4073-4075.1989 2472382 PMC210165

[42] Herzberg M, Bauer L, Nies DH. 2014. Deletion of the zupT gene for a zinc importer influences zinc pools in Cupriavidus metallidurans CH34. Metallomics 6:421. doi:10.1039/c3mt00267e 24407051

[43] Schmidt C, Schwarzenberger C, Große C, Nies DH. 2014. FurC regulates expression of zupT for the central zinc importer ZupT of Cupriavidus metallidurans. J Bacteriol 196:3461–3471. doi:10.1128/JB.01713-14 25049092 PMC4187674

[44] Huang DL, Tang DJ, Liao Q, Li HC, Chen Q, He YQ, Feng JX, Jiang BL, Lu GT, Chen BS, Tang JL. 2008. The Zur of Xanthomonas campestris functions as a repressor and an activator of putative zinc homeostasis genes via recognizing two distinct sequences within its target promoters. Nucleic Acids Res 36:4295–4309. doi:10.1093/nar/gkn328 18586823 PMC2490734

[45] Feng YJ, Li M, Zhang HM, Zheng BW, Han HM, Wang CJ, Yan JH, Tang JQ, Gao GF. 2008. Functional definition and global regulation of Zur, a zinc uptake regulator in a Streptococcus suis serotype 2 strain causing streptococcal toxic shock syndrome. J Bacteriol 190:7567–7578. doi:10.1128/JB.01532-07 18723622 PMC2576675

[46] Shin JH, Oh SY, Kim SJ, Roe JH. 2007. The zinc-responsive regulator Zur controls a zinc uptake system and some ribosomal proteins in Streptomyces coelicolor A3(2) . J Bacteriol 189:4070–4077. doi:10.1128/JB.01851-06 17416659 PMC1913400

[47] Maciag A, Dainese E, Rodriguez GM, Milano A, Provvedi R, Pasca MR, Smith I, Palù G, Riccardi G, Manganelli R. 2007. Global analysis of the Mycobacterium tuberculosis Zur (FurB) regulon. J Bacteriol 189:730–740. doi:10.1128/JB.01190-06 17098899 PMC1797298

[48] Outten CE, Tobin DA, Penner-Hahn JE, O’Halloran TV. 2001. Characterization of the metal receptor sites in Escherichia coli Zur, an ultrasensitive zinc(II) metalloregulatory protein. Biochemistry 40:10417–10423. doi:10.1021/bi0155448 11523983

[49] Patzer SI, Hantke K. 2000. The zinc-responsive regulator Zur and its control of the znu gene cluster encoding the ZnuABC zinc uptake system in Escherichia coli. J Biol Chem 275:24321–24332. doi:10.1074/jbc.M001775200 10816566

[50] Gaballa A, Helmann JD. 1998. Identification of a zinc-specific metalloregulatory protein, Zur, controlling zinc transport operons in Bacillus subtilis. J Bacteriol 180:5815–5821. doi:10.1128/JB.180.22.5815-5821.1998 9811636 PMC107652

[51] Bütof L, Große C, Lilie H, Herzberg M, Nies DH. 2019. Interplay between the Zur regulon components and metal resistance in Cupriavidus metallidurans. J Bacteriol 201:e00192-19. doi:10.1128/JB.00192-19 31109989 PMC6620404

[52] Bütof L, Schmidt-Vogler C, Herzberg M, Große C, Nies DH. 2017. The components of the unique Zur regulon of Cupriavidus metallidurans mediate cytoplasmic zinc handling. J Bacteriol 199:e00372-17. doi:10.1128/JB.00372-17 28808127 PMC5626952

[53] Edmonds KA, Jordan MR, Giedroc DP. 2021. COG0523 proteins: a functionally diverse family of transition metal-regulated G3E P-loop GTP hydrolases from bacteria to man. Metallomics 13:mfab046. doi:10.1093/mtomcs/mfab046 34302342 PMC8360895

[54] Haas CE, Rodionov DA, Kropat J, Malasarn D, Merchant SS, de Crécy-Lagard V. 2009. A subset of the diverse COG0523 family of putative metal chaperones is linked to zinc homeostasis in all kingdoms of life. BMC Genomics 10:470. doi:10.1186/1471-2164-10-470 19822009 PMC2770081

[55] Schulz V, Galea D, Herzberg M, Nies DH. 2024. Protecting the achilles heel: three FolE_I-type GTP-cyclohydrolases needed for full growth of metal-resistant Cupriavidus metallidurans under a variety of conditions. J Bacteriol 206:e0039523. doi:10.1128/jb.00395-23 38226602 PMC10882993

[56] Sankaran B, Bonnett SA, Shah K, Gabriel S, Reddy R, Schimmel P, Rodionov DA, de Crécy-Lagard V, Helmann JD, Iwata-Reuyl D, Swairjo MA. 2009. Zinc-independent folate biosynthesis: genetic, biochemical, and structural investigations reveal new metal dependence for GTP cyclohydrolase IB. J Bacteriol 191:6936–6949. doi:10.1128/JB.00287-09 19767425 PMC2772490

[57] Tanaka Y, Nakagawa N, Kuramitsu S, Yokoyama S, Masui R. 2005. Novel reaction mechanism of GTP cyclohydrolase I. High-resolution X-ray crystallography of Thermus thermophilus HB8 enzyme complexed with a transition state analogue, the 8-oxoguanine derivative. J Biochem 138:263–275. doi:10.1093/jb/mvi120 16169877

[58] Rebelo J, Auerbach G, Bader G, Bracher A, Nar H, Hösl C, Schramek N, Kaiser J, Bacher A, Huber R, Fischer M. 2003. Biosynthesis of pteridines. Reaction mechanism of GTP cyclohydrolase I. J Mol Biol 326:503–516. doi:10.1016/s0022-2836(02)01303-7 12559918

[59] Auerbach G, Herrmann A, Bracher A, Bader G, Gütlich M, Fischer M, Neukamm M, Garrido-Franco M, Richardson J, Nar H, Huber R, Bacher A. 2000. Zinc plays a key role in human and bacterial GTP cyclohydrolase I. Proc Natl Acad Sci USA 97:13567–13572. doi:10.1073/pnas.240463497 11087827 PMC17616

[60] Chandrangsu P, Huang X, Gaballa A, Helmann JD. 2019. Bacillus subtilis FolE is sustained by the ZagA zinc metallochaperone and the alarmone ZTP under conditions of zinc deficiency. Mol Microbiol 112:751–765. doi:10.1111/mmi.14314 31132310 PMC6736736

[61] Nies DH. 2019. The ancient alarmone ZTP and zinc homeostasis in Bacillus subtilis. Mol Microbiol 112:741–746. doi:10.1111/mmi.14332 31220391

[62] Gabriel SE, Miyagi F, Gaballa A, Helmann JD. 2008. Regulation of the Bacillus subtilis yciC gene and insights into the DNA-binding specificity of the zinc-sensing metalloregulator Zur. J Bacteriol 190:3482–3488. doi:10.1128/JB.01978-07 18344368 PMC2395016

[63] Cerasi M, Liu JZ, Ammendola S, Poe AJ, Petrarca P, Pesciaroli M, Pasquali P, Raffatellu M, Battistoni A. 2014. The ZupT transporter plays an important role in zinc homeostasis and contributes to Salmonella enterica virulence. Metallomics 6:845–853. doi:10.1039/c3mt00352c 24430377 PMC3969385

[64] Grass G, Wong MD, Rosen BP, Smith RL, Rensing C. 2002. ZupT is a Zn(II) uptake system in Escherichia coli. J Bacteriol 184:864–866. doi:10.1128/JB.184.3.864-866.2002 11790762 PMC139533

[65] Sharma R, Rensing C, Rosen BP, Mitra B. 2000. The ATP hydrolytic activity of purified ZntA, a Pb(II)/Cd(II)/Zn(II)-translocating ATPase from Escherichia coli. J Biol Chem 275:3873–3878. doi:10.1074/jbc.275.6.3873 10660539

[66] Rensing C, Mitra B, Rosen BP. 1997. The zntA gene of Escherichia coli encodes a Zn(II)-translocating P-type ATPase. Proc Natl Acad Sci U S A 94:14326–14331. doi:10.1073/pnas.94.26.14326 9405611 PMC24962

[67] Weast RC. 1984. CRC handbook of chemistry and physics. 64 ed. CRC Press, Inc, Boca Raton, Florida, USA.

[68] Liu JB, Dutta SJ, Stemmler AJ, Mitra B. 2006. Metal-binding affinity of the transmembrane site in ZntA: implications for metal selectivity. Biochem 45:763–772. doi:10.1021/bi051836n 16411752

[69] Tsai KJ, Lin YF, Wong MD, Yang HHC, Fu HL, Rosen BP. 2002. Membrane topology of the p1258 CadA Cd(II)/Pb(II)/Zn(II)-translocating P-type ATPase. J Bioenerg Biomembr 34:147–156. doi:10.1023/a:1016085301323 12171064

[70] González-Guerrero M, Argüello JM. 2008. Mechanism of Cu^+^-transporting ATPases: soluble Cu^+^ chaperones directly transfer Cu^+^ to transmembrane transport sites. Proc Natl Acad Sci U S A 105:5992–5997. doi:10.1073/pnas.0711446105 18417453 PMC2329688

[71] Argüello JM, Eren E, González-Guerrero M. 2007. The structure and function of heavy metal transport P1B-ATPases. Biometals 20:233–248. doi:10.1007/s10534-006-9055-6 17219055

[72] Nies DH, Silver S. 1989. Plasmid-determined inducible efflux is responsible for resistance to cadmium, zinc, and cobalt in Alcaligenes eutrophus. J Bacteriol 171:896–900. doi:10.1128/jb.171.2.896-900.1989 2914875 PMC209680

[73] Nies DH, Nies A, Chu L, Silver S. 1989. Expression and nucleotide sequence of a plasmid-determined divalent cation efflux system from Alcaligenes eutrophus. Proc Natl Acad Sci U S A 86:7351–7355. doi:10.1073/pnas.86.19.7351 2678100 PMC298059

[74] Grosse C, Anton A, Hoffmann T, Franke S, Schleuder G, Nies DH. 2004. Identification of a regulatory pathway that controls the heavy-metal resistance system Czc via promoter czcNp in Ralstonia metallidurans. Arch Microbiol 182:109–118. doi:10.1007/s00203-004-0670-8 15340798

[75] Grosse C, Grass G, Anton A, Franke S, Santos AN, Lawley B, Brown NL, Nies DH. 1999. Transcriptional organization of the czc heavy-metal homeostasis determinant from Alcaligenes eutrophus. J Bacteriol 181:2385–2393. doi:10.1128/JB.181.8.2385-2393.1999 10198000 PMC93662

[76] van der Lelie D, Schwuchow T, Schwidetzky U, Wuertz S, Baeyens W, Mergeay M, Nies DH. 1997. Two-component regulatory system involved in transcriptional control of heavy-metal homoeostasis in Alcaligenes eutrophus. Mol Microbiol 23:493–503. doi:10.1046/j.1365-2958.1997.d01-1866.x 9044283

[77] Smith AT, Ross MO, Hoffman BM, Rosenzweig AC. 2017. Metal selectivity of a Cd-, Co-, and Zn-transporting P-1B-type ATPase. Biochem 56:85–95. doi:10.1021/acs.biochem.6b01022 28001366 PMC5240476

[78] Schneider CA, Rasband WS, Eliceiri KW. 2012. NIH Image to ImageJ: 25 years of image analysis. Nat Methods 9:671–675. doi:10.1038/nmeth.2089 22930834 PMC5554542

[79] Herzberg M, Schüttau M, Reimers M, Große C Schlegel HG Nies DH. 2015. Synthesis of nickel-iron hydrogenase in Cupriavidus metallidurans is controlled by metal-dependent silencing and un-silencing of genomic islands. Metallomics 7:632–649. doi:10.1039/c4mt00297k 25720835

[80] Wiesemann N, Mohr J, Grosse C, Herzberg M, Hause G, Reith F, Nies DH. 2013. Influence of copper resistance determinants on gold transformation by Cupriavidus metallidurans strain CH34. J Bacteriol 195:2298–2308. doi:10.1128/JB.01951-12 23475973 PMC3650554

