# Supplementary material for: The efflux system CdfX exports zinc that cannot be transported by ZntA in Cupriavidus metallidurans
Source: J Bacteriol. 2024 Oct 30;206(11):e00299-24. doi: 10.1128/jb.00299-24 (PMC11580412; doi:10.1128/jb.00299-24)
Supplement: Supplemental figures and tables — Fig. S1 to S7; Tables S1 and S2. [file jb.00299-24-s0001.pdf]

## Supplementary material

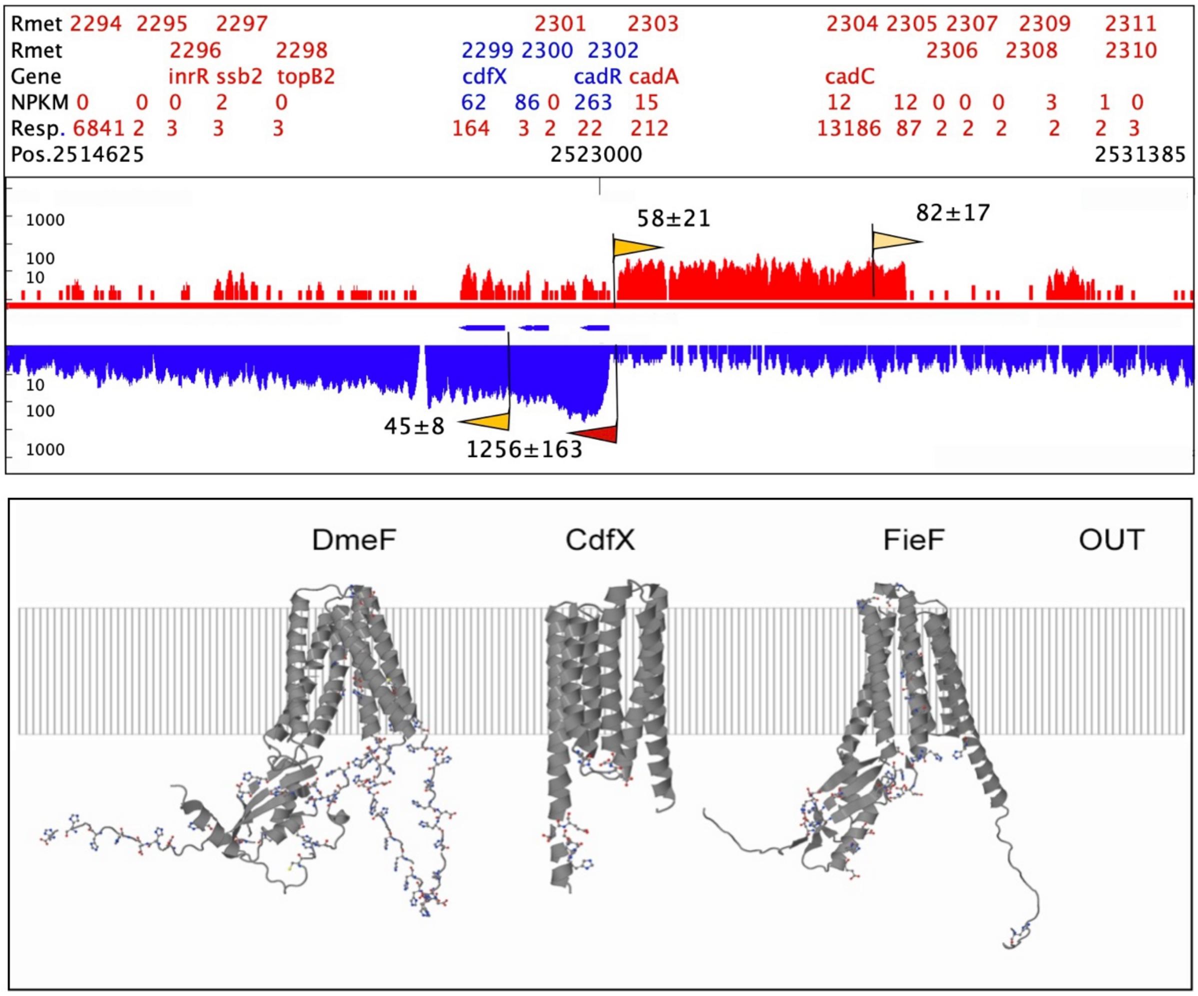


**Supplementary Figure S1. Maps of the *cad* determinant.** The map on the top shows the *cad* determinant including *cdfX* with NPKM ((nucleotide activities per kilobase of exon model per million mapped reads, a measure of transcript abundance) values on one DNA strand (red) or the other direction of transcription (blue). Above are the Rmet locus and gene names, the mean NPKM and response values (1). TSSs (flags) are indicated with the corresponding TSS score (not: RpoD score), red shades for strong (>1000, red) and orange for weak RpoD-dependent promoters. The RpoD-dependent promoter upstream of *cadR* is also deleted in all Δ*cadA* mutants (2). Below are AlphaFold2-derived structural models (3) of the cation ion diffusion facilitators (CDF) DmeF, CdfX and FieF for comparisons. His-rich sequences with negatively charged amino acyl residues are indicated.

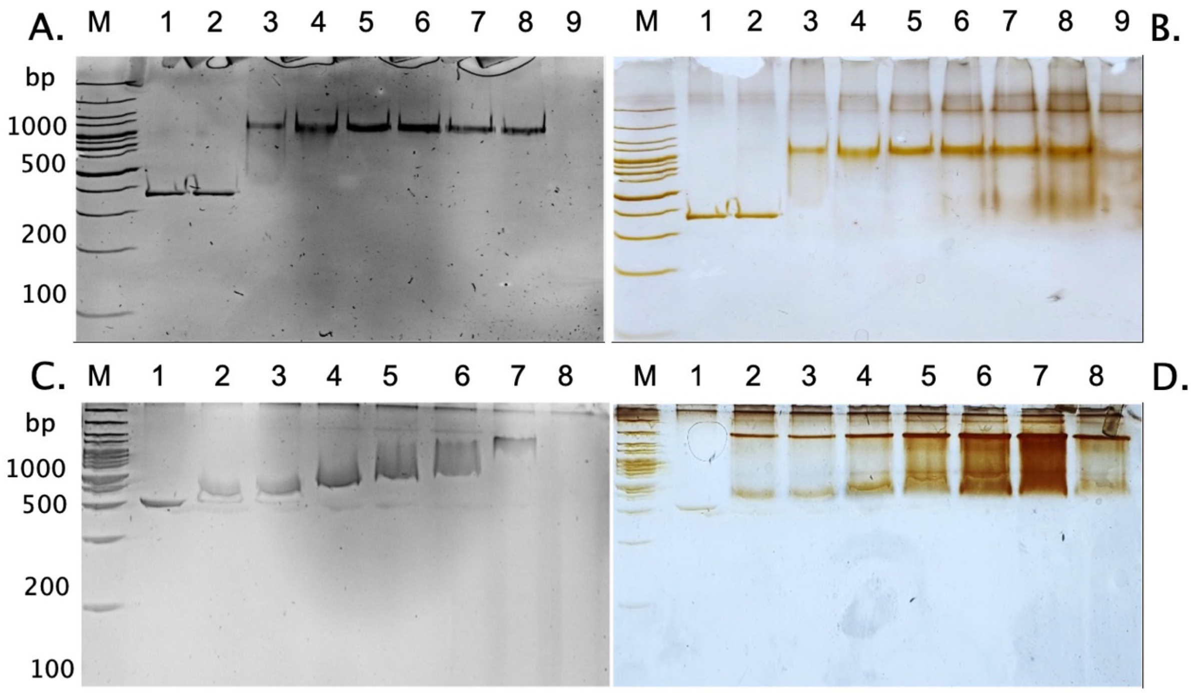


**Supplementary Figure S2. Electromobility shift assay (EMSA) with the ZntR protein and promoter regions of *zntR* and *cdfX*.** EMSAs were performed with purified ZntR protein containing 0.4 Zn atoms per polypeptide (4) and 400 fmol DNA containing the promoter regions *zntRp* (Panels A and B) or *cdfXp* (C and D). Lanes M, marker with indicated sizes in base pairs; 1, no protein. For *zntRp* (upper row), lanes 2 to 8 with 10, 20, 50, 100, 250, 400 and 500 pmol ZntR, respectively, and lane 9 without DNA but 250 pmol ZntR. For *cdfXp* (lower row), lanes 2 to 7 with 50, 100, 150, 200, 300 and 400 pmol ZntR, respectively, and 8 with no DNA but 200 pmol ZntR. HD green staining in Panels A and C, silver staining in Panels B and D. Controls were published (4).

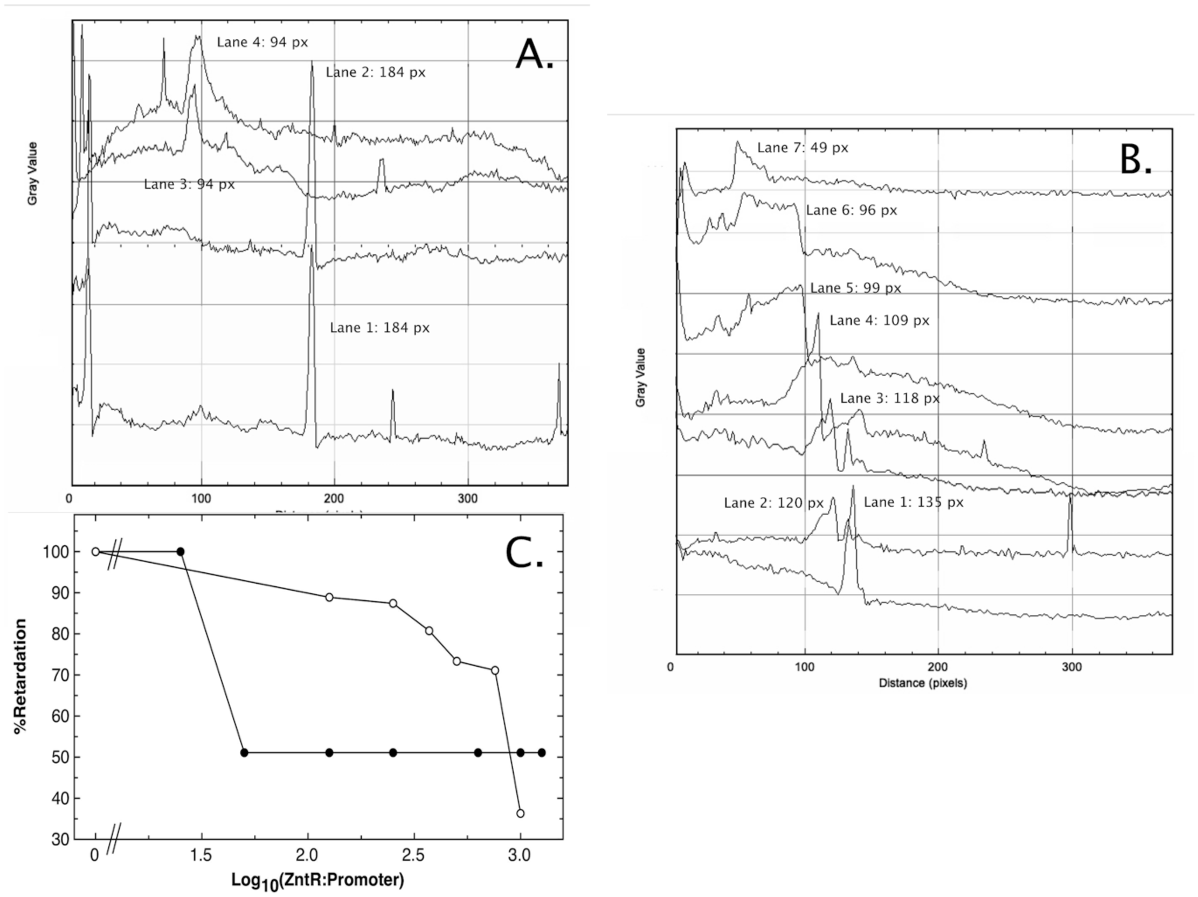


**Supplementary Figure S3. Detailed analysis of the EMSA experiments.** The lanes of the EMSA experiments (Suppl. Fig. S2) were analyzed with ImageJ (5) and the gray values of lanes 1 – 4 (Panel A, Suppl. Fig. S2, here Panels A and B, interaction of ZntR with *zntRp*) and 1 – 8 (Panel B, Suppl. Fig. S2, here Panels C and D, interaction of ZntR with *cdfXp*) are shown. The distance (in pixel) of the strongest intensity peak from the top of the gel was determined. These data were used to calculate the percent retardation, which is the distance of the peak in any lane divided by the distance of the peak in the respective lane 1. Panel C plots the percent retardation against the $Log_{10}$ of the ratio pmol ZntR to pmol promoter DNA of the *zntRp* (closed circles) or the *cdfXp* (open circles) promoter. It shows clearly a sigmoidal occupation of *zntRp* by ZntR with a 50% occupation between ZntR:*zntRp* ratios of 25 and 50. In contrast, *cdfXp* was only slowly occupied at higher ratios and 50% occupation occurs at a ration > 750. Since the DNA-protein complexes were formed at 30°C for 40 min in 30 µL binding buffer, this represents a ZntR-concentration required for 50% occupation of about 0.5 µM for *zntRp* and > 10 µM for *cdfXp*. Consequently, ZntR binds first to its own promoter *zntRp* and subsequently to *cdfXp*.

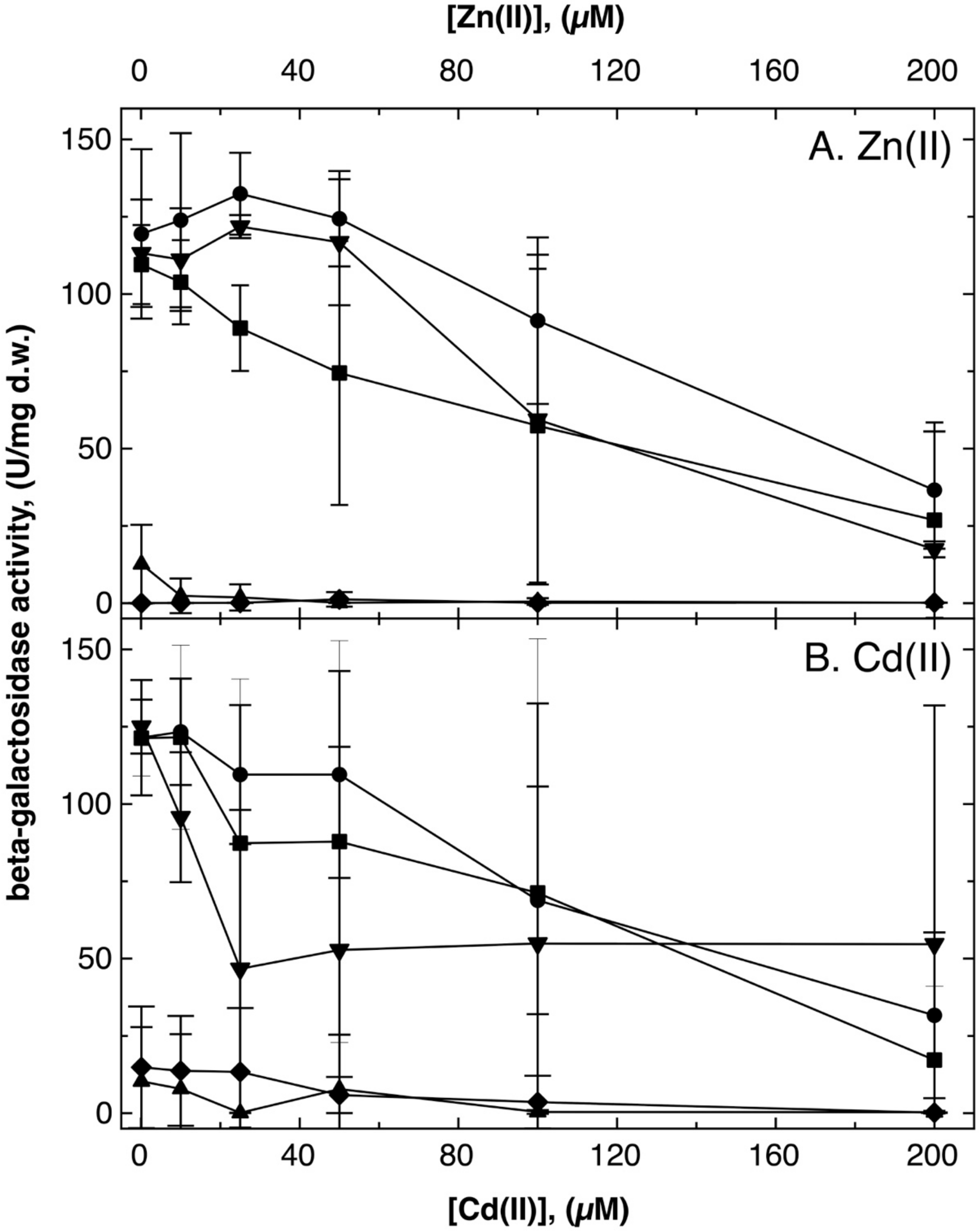


**Supplementary Figure S4. Regulation of *cadR* by zinc and cadmium as determined with a Φ*(cadR::lacZ*) reporter gene fusion in TMM.** The beta-galactosidase activity of a Φ(*cadR::lacZ*) transcriptional fusion in response to increasing zinc (Panel A) or cadmium (Panel B) concentrations was determined in strain AE104 (closed circles), Δ*zntA* (closed squares), Δ*cadA* (closed diamonds), Δ*zntA* Δ*cadA* (closed triangles), Δ*zntR* (closed inverted triangles), n >= 3, deviations shown.

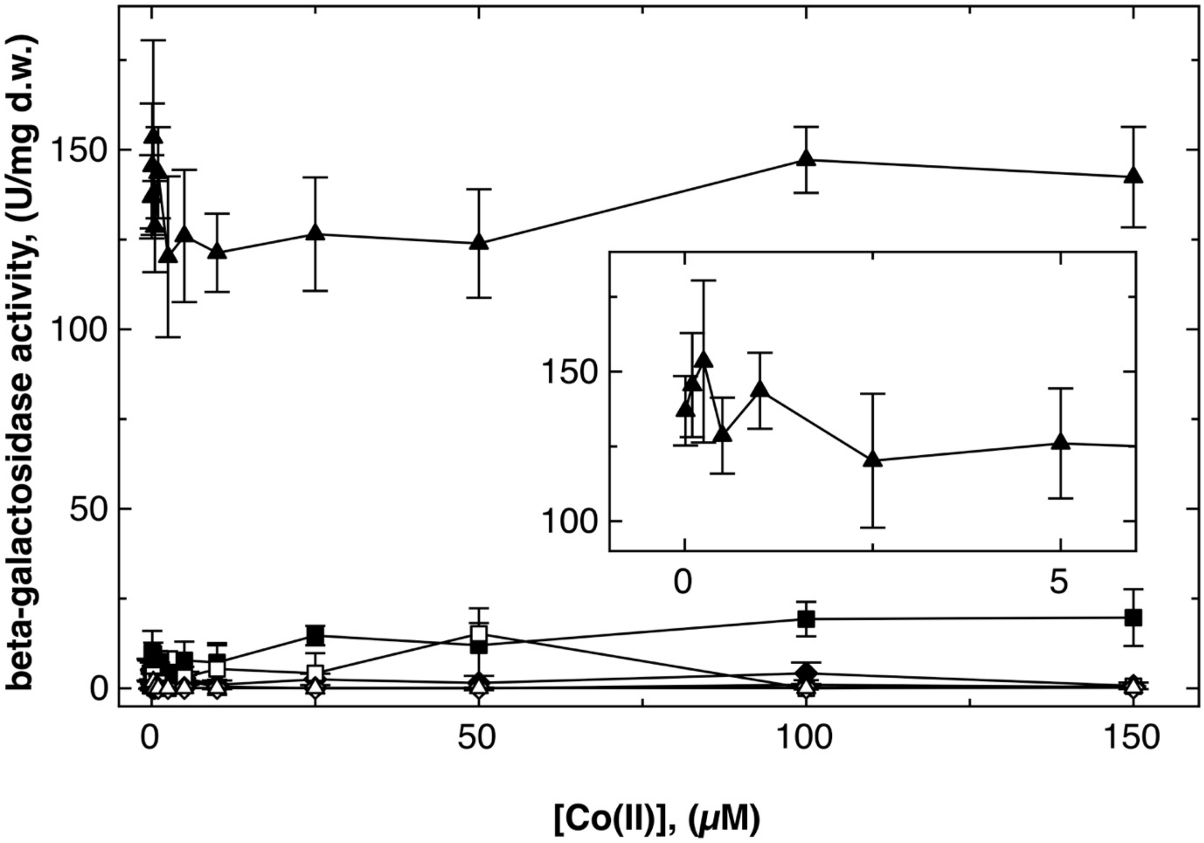


**Supplementary Figure S5. Regulation of *cdfX* by cobalt ions as determined with a Φ*(cdfX::lacZ*) reporter gene fusion in TMM.** The beta-galactosidase activity of a Φ(*cdfX::lacZ*) transcriptional fusion in response to increasing cobalt concentrations was determined in strain AE104 (closed circles), Δ*zntA* (closed squares), Δ*cadA* (closed diamonds), Δ*zntA* Δ*cadA* (closed triangles) and additional Δ*zntR* deletions in the strains Δ*zntA* (open squares), Δ*cadA* (open diamonds) and Δ*zntA* Δ*cadA* (open triangles). The inset gives the Δ*zntA* Δ*cadA* Φ(*cdfX::lacZ*) strain at low cobalt concentrations with a higher resolution, n = 3, deviations shown.

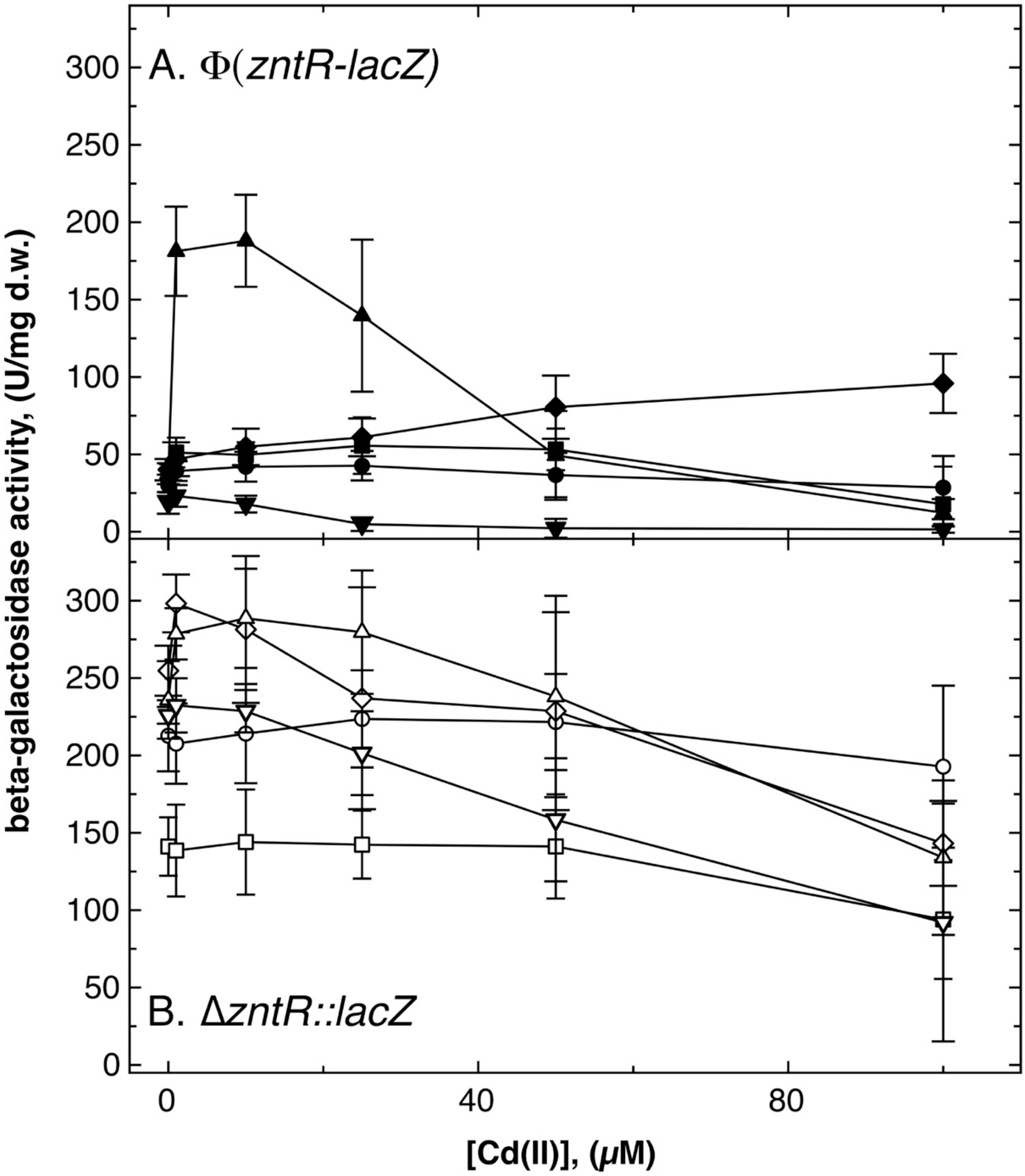


**Supplementary Figure S6. Regulation of *zntR* by cadmium as determined with a *zntR* - *lacZ* reporter gene fusion in TMM.** The beta-galactosidase activity of a *zntR-lacZ* transcriptional fusion in response to increasing cadmium concentrations was determined in strain AE104 (circles), Δ*zntA* (squares), Δ*cadA* (diamonds), Δ*zntA* Δ*cadA* (triangles) and Δ*cadR* (inverted triangles) deletion mutants. Panel A shows Φ(*zntR-lacZ*) fusion that did not affect *zntR* (closed symbols), Panel B fusions that interrupted *zntR* (open symbols), n >= 3, deviations shown.

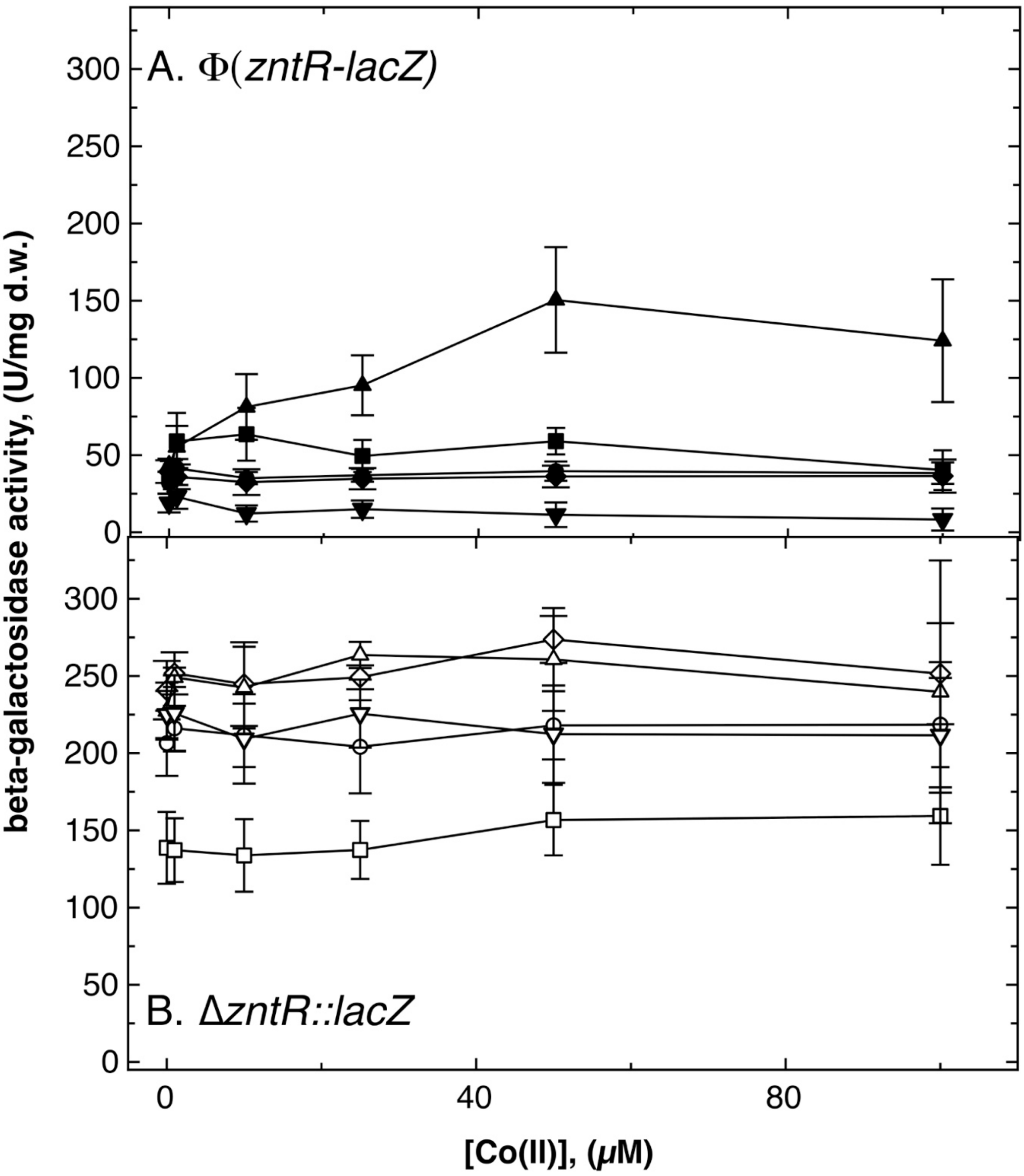


**Supplementary Figure S7. Regulation of *zntR* by cobalt as determined with a *zntR-lacZ* reporter gene fusion in TMM.** The beta-galactosidase activity of a *zntR-lacZ* transcriptional fusion in response to increasing cobalt concentrations was determined in strain AE104 (circles), Δ*zntA* (squares), Δ*cadA* (diamonds), Δ*zntA* Δ*cadA* (triangles) and Δ*cadR* (inverted triangles) deletion mutants. Panel A shows Φ(*zntR-lacZ*) fusion that did not affect *zntR* (closed symbols), Panel B fusions that interrupted *zntR* (open symbols), n >= 3, deviations shown.

**Supplementary Table S1. Minimal inhibitory concentrations (MIC) of strains with deletions in the genes for the regulators[a].**

| Strain | Zn(II) | Cd(II) | Co(II) |
|---|---|---|---|
| AE104 | 238±47 | 613±91 | 900±0 |
| Δ*zntA* | 142±11 | 500±57 | 850±67 |
| Δ*cadA* | 300±0 | 588±138 | 1167±111 |
| Δ*cadA*Δ*zntA* | 25.0±0.0 | 0.5±0.0 | 967±44 |
| | | | |
| Δ*zntR* | 142±11 | 750±0 | 1083±111 |
| Δ*cadR* | 267±44 | 750±0 | 1250±0 |
| Δ*cadR* Δ*zntR* | 142±11 | 567±67 | 850±67 |
| | | | |
| Δ*zntA* Δ*zntR::dis* | 117±11 | 443±94 | 850±67 |
| Δ*cadA* Δ*zntR::dis* | 50.0±0.0 | 0.8±0.3 | 1167±111 |
| Δ*cadA* Δ*zntA* Δ*zntR::dis* | 13.8±5.6 | 0.5±0.0 | 1250±0 |

[a]n ≥ 3. A preculture in TMM was diluted 100-fold with fresh TMM and 1 µL was spotted on TMM plates with increasing metal concentrations. The MIC was determined after 5 days of incubation at 30°C.

## Supplementary Table S2. Bacterial strains and primers

| Strain | Description | Reference |
|---|---|---|
| ***C. metallidurans*** | | |
| AE104 | Plasmid-free | (6) |
| DN595 | AE104Δ*zntA* | (7) |
| DNA45 | AE104ΔzntAΔzntR | this work |
| DN438 | AE104Δ*cadA* | (2) |
| DNA46 | AE104Δ*cadA*Δ*zntR* | this work |
| DNA47 | AE104Δ*zntA*Δ*cadA*Δ*zntR* | this work |
| DNA71 | AE104Δ*cdfX* | this work |
| DN600 | AE104Δ*cadA*Δ*zntA* | (7) |
| DN727 | AE104Δ*cadR* | (8) |
| DN781 | AE104Δ*zntR* | (8) |
| DN973 | AE104Δ*cadR*Δ*zntR* | this work |
| DN578 | AE104Δe4 (ΔzntAΔcadAΔfieFΔdmeF) | (7) |
| DNA73 | AE104Δe4ΔcdfX(ΔzntAΔcadAΔfieFΔdmeF) | this work |
| ***E. coli*** | | |
| ECB284 | S17-1 (pECD794.1 Φ(*cdfX-lacZ*) | this work |
| ECB286 | S17-1 (pECD1003::Δ*cdfX*) | this work |
| ECB049 | S17-1 with pECD1473 (pECD1002::Δ*zntR*) | (8) |
| ECB257 | S17-1 with pECD1675 (pECD794.1, Φ(*zntR-lacZ*)) | this work |
| ECB258 | S17-1 with pECD1676 (pECD794.1, Δ*zntR*::*lacZ*) | this work |
| ECB259 | S17-1 with pECD1677 (pECD794.1, Φ (*cadR-lacZ*) | this work |

| Primers | Sequence 5`→ 3` | Binding site |
|---|---|---|
| **Controls primers** | | |
| lacZ ralsti fusion test → | CACAGATGAAACGCCGAGTTAACG | test/sequencing primer for pECD794.1 vectors, binds 278 bp upstream of $ATG_{lacZ}$ |
| pLO2-lacZ-5519 ← | GGCGGAAAATCGTGTTGAGGC | test/sequencing primer for pECD794.1 vectors, binds binds 438 bp downstream of $ATG_{lacZ}$ |
| **Primers for EMSA** | | |
| 2299p SpeI → | AAAACTAGTAGCGAGCGCGACGATCAC | 293 bp upstream of $ATG_{cdfX}$ |
| 2299p KpnI ← | AAAGGTACCGCATTGTTCCTTCCAAATCGA | directly upstream of $ATG_{cdfX}$ |
| Rm3456_NcoI → | AAACCATGGCGAGCGCGTGCTTTACGATG | 332 bp upstream of $ATG_{zntR}$ |
| promo_zntR_rev_ApaI ← | AAAGGGCCCGGTCAGTGCCTCCAC | directly upstream of $ATG_{zntR}$ |
| **Primers for *lacZ*-fusion and disruption experiments** | | |
| rm3456-disr-SalI → | AAAGTCGACGACCATGCGTATCGGCGAA | 12 bp upstream and directly at $ATG_{zntR}$ |
| rm3456-disr-XbaI ← | AAATCTAGACGTGGATCTGCTCGATCTGG | 257 bp downstream of $ATG_{zntR}$ |
| rm 3456 lacZ SalI → | AAAGTCGACTCGTCCAGCTCAACTTCGTG | 129 bp downstream of $ATG_{zntR}$ |
| rm 3456 Stop-XbaI ← | GGGTCTAGACTAGTGTCGTGGGTGGCACTC | Directly upstream of and at $TGA_{zntR}$ |
| AL cadR lacZ 3' PstI → | AAACTGCAGGGTGAACTGGGCAAGAAGGCA | ca. 10 bp downstream of Start-Codon $ATG_{Rmet\_2302}$ |

| | | |
|---|---|---|
| 2302_XbaI_rev ← | AAATCTAGATCAGGCGGGCTCGGCCAAG | directly upstream of and at $TGA_{cadR}$ |
| 2299 Dis 1 Sph | GCATGCAGATGCGAGCGATGCGAAG | For *cdfX-lacZ* |
| 2299 Dis 2 Xba | TCTAGATGGGCCGACAGGCTCTTC | For *cdfX-lacZ* |
| cdfX Mun 671 up | AAACAATTGCTCTAGCAAGGCGGCACAAGC | For Δ*cdfX*, pos 671 |
| cdfX Not 998 down | AAAGCGGGCCGCTCTGTGGTTTTCAGCCATGCA | For Δ*cdfX*, pos 998 |
| cdfX Apa 1641 up | AAAGGGCCCTGTTTTCGCCCAGTTCATCCC | For Δ*cdfX*, pos 1641 |
| cdfX Age 1973 down | AAAACCGGTCCGGGTGTGATTGCTTGATGA | For Δ*cdfX*, pos 1973 |

**References of the Supplement**


1. Große C, Kohl T, Herzberg M, Nies DH. 2022. Loss of mobile genomic islands in metal resistant, hydrogen-oxidizing *Cupriavidus metallidurans*. Appl Environ Microbiol 88:e02048-21.
2. Legatzki A, Anton A, Grass G, Rensing C, Nies DH. 2003. Interplay of the Czc-system and two P-type ATPases in conferring metal resistance to *Ralstonia metallidurans*. J Bacteriol 185:4354–4361.
3. Varadi M, Anyango S, Deshpande M, Nair S, Natassia C, Yordanova G, Yuan D, Stroe O, Wood G, Laydon A, Žídek A, Green T, Tunyasuvunakool K, Petersen S, Jumper J, Clancy E, Green R, Vora A, Lutfi M, Figurnov M, Cowie A, Hobbs N, Kohli P, Kleywegt G, Birney E, Hassabis D, Velankar S. 2021. AlphaFold Protein Structure Database: massively expanding the structural coverage of protein-sequence space with high-accuracy models. Nucleic Acids Research 50:D439-D444.
4. Schulz V, Schmidt-Vogler C, Strohmeyer P, Weber S, Kleemann D, Nies DH, Herzberg M. 2021. Behind the shield of Czc: ZntR controls expression of the gene for the zinc-exporting P-type ATPase ZntA in *Cupriavidus metallidurans*. J Bacteriol 203:e00052-21.
5. Schneider CA, Rasband WS, Eliceiri KW. 2012. NIH image to ImageJ: 25 years of image analysis. Nat Methods 9:671-675.
6. Mergeay M, Nies D, Schlegel HG, Gerits J, Charles P, van Gijsegem F. 1985. *Alcaligenes eutrophus* CH34 is a facultative chemolithotroph with plasmid-bound resistance to heavy metals. J Bacteriol 162:328-334.
7. Scherer J, Nies DH. 2009. CzcP is a novel efflux system contributing to transition metal resistance in *Cupriavidus metallidurans* CH34. Mol Microbiol 73:601-621.
8. Schmidt C, Schwarzenberger C, Grosse C, Nies DH. 2014. FurC regulates expression of *zupT* for the central zinc importer ZupT of *Cupriavidus metallidurans*. J Bacteriol 196:3461-3471.
